# Influence of the Extraction Method on the Polyphenolic Profile and the Antioxidant Activity of *Psidium guajava* L. Leaf Extracts

**DOI:** 10.3390/molecules29010085

**Published:** 2023-12-22

**Authors:** Daniela Gutierrez Montiel, Alma Lilian Guerrero Barrera, Guillermo Cristian Guadalupe Martínez Ávila, María Dolores Gonzalez Hernandez, Norma Angelica Chavez Vela, Francisco Javier Avelar Gonzalez, Flor Yazmin Ramírez Castillo

**Affiliations:** 1 Laboratorio de Biología Celular y Tisular, Departamento de Morfología, Centro de Ciencias Básicas, Universidad Autónoma de Aguascalientes, Aguascalientes CP 20100, Mexico; al158823@edu.uaa.mx (D.G.M.); flor.ramirez@edu.uaa.mx (F.Y.R.C.); 2 Laboratorio de Química y Bioquímica, Facultad de Agronomía, Universidad Autónoma de Nuevo León, General Escobedo CP 66050, Mexico; lolis.90.6@gmail.com; 3 Laboratorio de Biotecnología, Departamento Ingeniería Bioquímica, Centro de Ciencias Básicas, Universidad Autónoma de Aguascalientes, Aguascalientes CP 20100, Mexico; angelica.chavez@edu.uaa.mx; 4 Laboratorio de Estudios Ambientales, Departamento de Fisiología y Farmacología, Centro de Ciencias Básicas, Universidad Autónoma de Aguascalientes, Aguascalientes CP 20100, Mexico; fjavelar@correo.uaa.mx

**Keywords:** *Psidium guajava* L., polyphenolics, antioxidants, characterization, phytochemicals, guava leaves, UPLC-MS

## Abstract

The leaves of *Psidium guajava* L. are an agro-industrial by-product with an outstanding content of polyphenolic compounds; however, there are many factors which can affect the phytochemical profile when valuing this type of plant material, such as temperatures and extraction times involving in the extraction methods applied. In this context, this study analyzed the impact of different extraction methods (Soxhlet, maceration and ultrasound-assisted extraction) on the phytochemical profile (FTIR and UPLC-MS) and the antioxidant activity (ABTS, FRAP and Folin–Ciocalteu) of guava leaf extracts. A yield of phenolic compounds per gram of guava leaf was obtained within the range of 16 to 45 mg/g; on the other hand, the ${IC}_{50}$ values determined with the ABTS assay ranged between 78 ± 4 to 152 ± 12 µg/mL. The methanolic extract obtained by Soxhlet was the one with the best reducing power, both in the FRAP assay and in the Folin–Ciocalteu assay. Finally, bioactive compounds such as quercetin, kaempferol and avicularin were identified in the guava leaf extract. It was concluded that the purification of polyphenolics compounds improves the antioxidant capacity, and that the extraction method greatly influences the phytochemical profile and activity of the extracts.

## 1. Introduction

Recently, there has been a growing interest from the scientific community in phytochemicals, non-nutritive bioactive compounds of plant origin, which have beneficial properties for health, as well as great antioxidant power, which makes them potential nutraceuticals, products that are able to prevent diseases and thereby increasing its value for the society [1].

One of the most abundant groups of phytochemicals are polyphenolic compounds, which are secondary plant metabolites having highly varied structures but are characterized by the presence of aromatic rings and hydroxyl groups [2]. Polyphenolic compounds are recognized as antioxidants for their ability to donate hydrogen atoms and/or electrons to free radicals, breaking the oxidation chain [3]. This ability has become a topic of great importance considering that oxidative stress has been associated with many diseases, such as cancer, hypertension, diabetes mellitus, atherosclerosis and neurological disorders [3].

Oxidative stress occurs when there is no balance between pro-oxidant species and antioxidants [4]. It should be noted that oxidation is not a specific problem for humans, it also appears in food, e.g., the oxidation of lipids and proteins, which is a serious problem, as it can cause the destruction of essential nutrients, bad odors and even the generation of toxic compounds in food systems [5].

Downstream processing involves essential steps in discovering bioactive compounds from raw plant materials, including the extraction methods. Different factors such as solvent type, temperature, time and the solid–liquid ratio affect the extraction efficiency, so they must be carefully selected, considering the technique to be used [6]. The most popular techniques are maceration, infusion and continuous hot extraction, such as Soxhlet; however, alternative methods such as ultrasonic or microwave-assisted extraction and supercritical fluid extractions have become available, and have gained interest because they are faster and, above all, respectful of the environment by reducing solvent and energy consumption [7].

Each extraction technique has its own advantages and disadvantages, in the case of Soxhlet extraction, it allows to extract a large quantity and variety of compounds in a relatively short time; furthermore, the solvent can be reused and the filtration and/or centrifugation process for separation the plant material can be avoided. However, generally, high temperatures are used, and, consequently, the degradation of phytochemicals, including polyphenolic compounds, may occur [6]. Therefore, in this study, we evaluated other options which did not require high temperatures: maceration due to its simplicity and wide use [8] and ultrasound-assisted extraction that facilitates the breaking of plant cells, increasing the contact area between plant material and solvent, decreasing heat requirements [6]. It is important to highlight that the extraction of polyphenolic compounds is a challenge since they can be unstable and the biological activity can be damaged and/or lost by high temperatures and the presence of oxygen and light; therefore, the choice of the extraction technique and all its parameters is key to obtain good yields and at the same time maintain the integrity of the compounds [9].

In this sense, *Psidium guajava* L. is a native American shrub of great economic importance for its fruit, the guava, a berry with firm pulp and numerous seeds that is marketed worldwide. The harvest *P. guajava* generates a large number of leaves as by-products, which are rich in polyphenolic compounds, and it has also been reported that they have antimicrobial and antioxidant activity [10]. Unfortunately, about 1.3 billion tons of agricultural by-products, including leaves, seeds and skins are wasted each year [2] and are landfilled or incinerated even though they may contain large amounts of bioactive compounds. These practices only increase the environmental load and the total cost of production [11,12]. In the specific case of guava, approximately 80 kg of this by-product per metric ton of fresh fruit is produced during guava processing [13].

The valorization of guava leaves is an opportunity for producing countries such as Mexico, India and China, where large amounts of leaves are available [8]. However, for its use, it is necessary to consider that, depending on the geographical location, weather conditions, the presence of different pathogens, the variety, etc., changes can occur in the phytochemical composition of the plant organs [14,15], so it is extremely important to consider the parts of the plant material.

In the present study, the impact of different extraction methods on the antioxidant activity and the phytochemical profile of guava leaf extracts was evaluated, considering two conventional techniques and a green alternative. In addition, to our best knowledge, this is the first report in which the polyphenolic compounds of the guava leaf are purified with amberlite XAD-16, achieving improvements in their bioactivity.

## 2. Results

The leaves of *P. guajava* L. are recognized for their high and diverse content of polyphenolic compounds [16,17,18], as well as for their wide use in traditional medicine to treat conditions such as stomach pains, wounds, cavities and coughs [10]. In recent decades, evidence has demonstrated the beneficial properties of polyphenols, for example, their antioxidant, antimicrobial and anti-inflammatory activity [3,19]. The extraction of polyphenolic compounds from agro-industrial residues is a viable option to value this type of waste, minimizing environmental damage and generating value-added products, such as natural antioxidants in functional foods or for the development of dietetic supplements [19]. This section presents the tentative identification of the polyphenolic compounds present in guava leaf extracts as well as their antioxidant activity, discussing the differences observed according to the extraction method used, which consists of a given technique, temperature and time.

### 2.1. Purification of Polyphenolic Compounds

Table 1 shows the different extraction methods used and the yields of polyphenolic compounds obtained using them, which ranged from 16 to 45 mg·g^−1^. The highest values were observed with the extraction method of Soxhlet, using methanol as an extracting solvent (44 mg·g^−1^), and ultrasound-assisted extraction at 30 °C with distilled water (45 mg·g^−1^). On the other hand, all other extraction methods had an approximate yield of around 20 mg·g^−1^.

Multiple authors have reported different yields of total polyphenolic compounds in guava leaves. For example, Farag et al. [20] observed a total content of 59.267 ± 0.348 mg GAE (gallic acid equivalent) ·g^−1^; Sowmya et al. [21] reported a content of 41.33 ± 0.92 mg GAE·g^−1^ and 37.60 ± 0.26 mg GAE·g^−1^ in two guava varieties; and Laily et al. [22] published a content of 101.93 mg GAE·g^−1^. Although these concentrations may be higher than those obtained in this study, it is important to mention that, in the works mentioned, a purification process for polyphenolic compounds was not carried out, so the total content may be overestimated, especially considering that the Folin–Ciocalteu assay was used for the determination. Even if this assay is well established and widely used, it should be noted that since it is based on a redox reaction, compounds other than phenols, for example, reducing sugars and ascorbic acid, can also reduce the Folin–Ciocalteu reagent [23]. It is worth mentioning that the yield is not directly related to the phenolic composition of the samples or to their antioxidant activity; therefore, a greater number of polyphenolic compounds does not always mean better antioxidant activity [24].

### 2.2. Antioxidant Activity

The antioxidant activity was evaluated using three different assays: Folin–Ciocalteu and FRAP (ferric-reducing antioxidant power), both to analyze the reducing capacity of the samples, and ABTS (2,2′-Azino-bis (3-ethylbenzthiazoline-6-sulfonic acid)), to analyze the ability to inhibit the radical cation ABTS^•+^.

**Figure 1:**
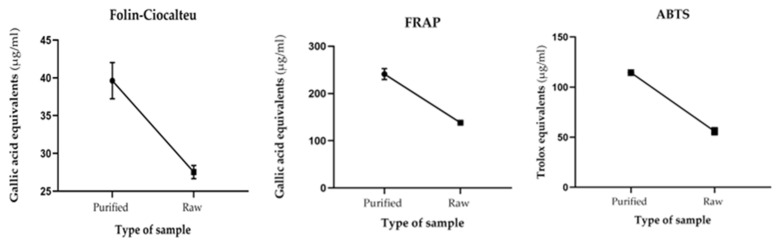
Mean and SEM of crude and purified samples in different antioxidant capacity assays.

Preliminarily, the three tests (Folin–Ciocalteu, FRAP and ABTS^•+^) were carried out with both the crude and purified solutions of the guava leaf extracts at 0.250 mg·mL^−1^, and it was observed that in all the tests, the samples with purified polyphenolics had a higher antioxidant activity in comparison with the raw samples, as shown in Figure 1.

This behavior is especially interesting given that Folin–Ciocalteu and FRAP are not specific tests for polyphenols, since they can be reduced by other agents such as reducing sugars, amino acids and ascorbic acid [25], which could be present in raw samples [26]. For this reason, it is important to carry out a purification process, since it allows us to eliminate inert and undesirable components that can interfere with the study and/or that have limited antioxidant activity that represses the activity of polyphenols [27].

The determination of the ${IC}_{50}$ (the minimum extract concentration at which 50% of the free radicals are inhibited) values were carried out with the ABTS^•+^ assay. The results are within the range from 78 to 152 µg·mL^−1^, as shown in Table 2. It is worth mentioning that the lowest values of ${IC}_{50}$ indicate a greater capacity to inhibit free radicals of the samples, which in the present study were exhibited by the methanolic extracts obtained by maceration and ultrasound (87 to 78 µg·mL^−1^).

Other studies with guava leaves have reported lower ${IC}_{50}$ values: 24.37 ± 3.85 μg·mL^−1^ [28], 31.19 ± 5.01 to 72.31 ± 3.57 μg·mL^−1^ [29] and 3.23 ± 0.24 to 8.26 ± 1.06 μg·mL^−1^ [30]. This may be since the polyphenol content and, therefore, its antioxidant activity may be affected by different factors, including the extraction conditions (technique, temperature, solvent, time, etc.), climatic conditions and soil quality [14,15,31]. Even so, guava leaf collected in Aguascalientes, Mexico presented relevant antioxidant activity compared to other plant extracts characterized by exhibiting different functional properties such as avocado leaf extracts (${IC}_{50}$ = 269.56 ± 6.52 to 442.72 ± 9.62 μg·mL^−1^) [32], rosemary leaf extracts (${IC}_{50}$ = 70 ± 4.67 μg·mL^−1^) [33] and oriental ebony leaf extracts (${IC}_{50}$ = 108.7 μg·mL^−1^) [34].

Similarly, it has been observed that guava leaves have a concentration of polyphenolic compounds and an antioxidant capacity higher than other parts of the bush such as seeds, fruit and bark [30,35]; consequently, the present study shows that guava leaf extracts can be considered an alternative for obtaining bioactive compounds with multiple applications in industry.

**Figure 2:**
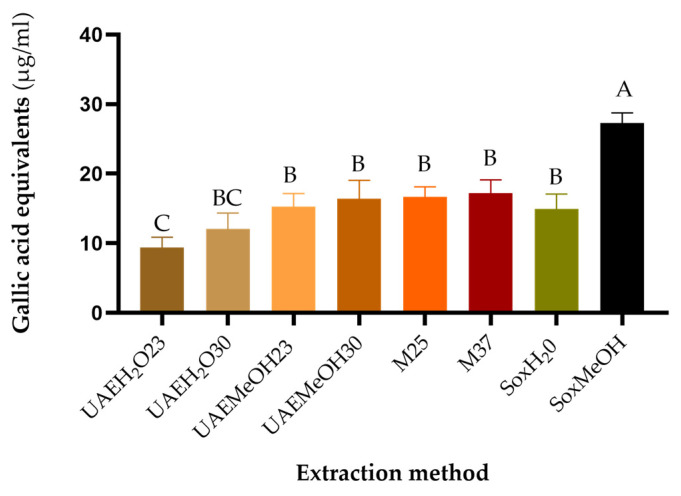
Folin–Ciocalteu reagent reducing capacity according to the extraction method. SoxMeOH: Soxhlet, methanol, 65 °C, 5 h; Sox$H_{2}O$: Soxhlet, distilled water, 100 °C, 5 h; M37: maceration, methanol, 37 °C, 192 h; M25: maceration, methanol, 25 °C, 192 h; UAEMeOH30: ultrasound, methanol, 30 °C, 0.66 h; UAEMeOH23: ultrasound, methanol, 23 °C, 0.66 h; UAE$H_{2}O$30: ultrasound, distilled water, 30 °C, 0.66 h; UAE$H_{2}O$23: ultrasound, distilled water, 23 °C, 0.66 h. Methods that do not share a letter are significantly different (*p* < 0.05).

**Figure 3:**
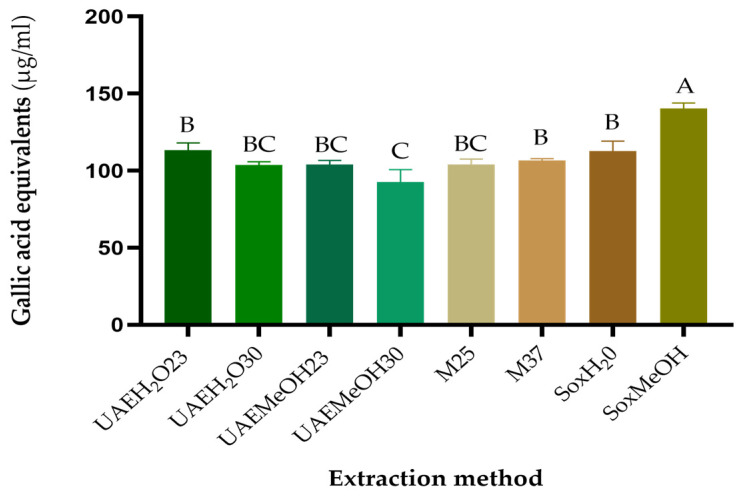
Iron-reducing capacity according to the extraction method. SoxMeOH: Soxhlet, methanol, 65 °C, 5 h.; Sox$H_{2}O$: Soxhlet, distilled water, 100 °C, 5 h.; M37: maceration, methanol, 37 °C, 192 h; M25: maceration, methanol, 25 °C, 192 h; UAEMeOH30: ultrasound, methanol, 30 °C, 0.66 h; UAEMeOH23: ultrasound, methanol, 23 °C, 0.66 h; UAE$H_{2}O$30: ultrasound, distilled water, 30 °C, 0.66 h; UAE$H_{2}O$23: ultrasound, distilled water, 23 °C, 0.66 h. Methods that do not share a letter are significantly different (*p* < 0.05).

The FRAP and Folin–Ciocalteu assays were performed with the purified guava leaf extracts at respective concentrations of ${IC}_{50}$ (Table 1). In both tests, it was observed that the extract with the highest reducing capacity was the one obtained using the Soxhlet extraction method with methanol as the extracting solvent, while the other extracts exhibited a similar reducing capacity, as can be seen in Figure 2 and Figure 3.

Temperature is an important factor for the extraction of phytochemicals [8]. Increases in temperature can result in improvements in extraction thanks to better diffusion and solubilization [36,37]; however, they could also generate the degradation of compounds and decrease antioxidant activity [38,39]. In our case, the extraction method with Soxhlet is the one that used the highest temperature (65 °C), and this favored the yield of polyphenolic compounds and antioxidant activity, specifically the reducing activity.

In recent years, the search for natural antioxidants has gained importance in view of the need to replace fossil-derived resources, as well as to avoid the use of synthetic antioxidants, which can be cytotoxic and carcinogenic [40,41]. The different guava leaf polyphenolic extracts studied exhibited good antioxidant activity, property useful in a wide variety of applications, such as in the food [42], cosmetic [43,44] and pharmaceutical industries [45], meaning they could be a low-cost alternative of natural origin. For example, the methanolic extract obtained by Soxhlet, on account of its significant reducing capacity, may be a good candidate for the green synthesis of nanoparticles [46,47], while the methanolic extracts obtained by maceration and ultrasound-assisted extraction may be good candidates as nutraceuticals or cosmetics given their ability to inhibit free radicals [48]. Obviously, previous studies on toxicity, stability and bioavailability as well as a solvent removal process are necessary before the extract can have an industrial application.

### 2.3. Characterization by FTIR

The dried purified methanolic extracts obtained by three different methods (Soxhlet, maceration at 25 °C and ultrasound-assisted extraction at 30 °C) were selected due to their notable results in antioxidant activity and yield in polyphenolic compounds for analysis by Fourier transform infrared spectroscopy (FTIR). It should be noted that, as the samples analyzed are purified extracts, the peaks of the spectra correspond to the different functional groups present in the polyphenolic compounds.

**Figure 4:**
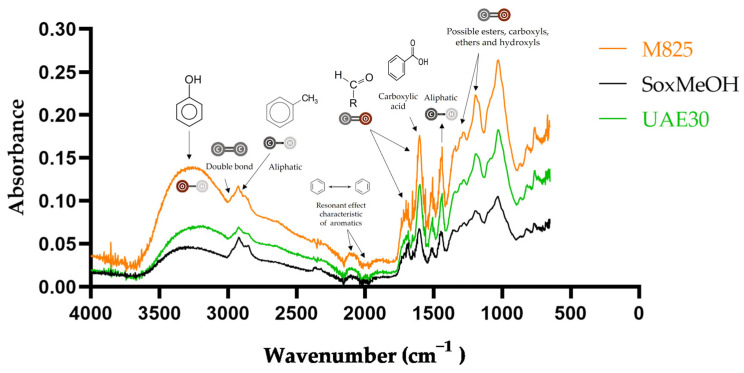
FTIR analysis of the methanolic extracts of guava leaves. M825: maceration at 25 °C; SoxMeOH: Soxhlet at 65 °C; UAE30: ultrasound-assisted extraction at 30 °C.

In Figure 4, from 3000 to 3600 ${cm}^{-1}$, the broad and strong band corresponds to the stretching vibrations of the O–H bond, which indicates the presence of functional groups such as hydroxyls. In addition, the presence of O–H groups is confirmed since there is vibration between 1600 and 1300 ${cm}^{-1}$, 1200 and 1000 ${cm}^{-1}$ and 800 and 600 ${cm}^{-1}$. The presence of peaks from 3000 to 2900 ${cm}^{-1}$ corresponds to the stretching of the C–H bond, which is characteristic of aliphatic functional groups. On the other hand, from 2200 to 1950 ${cm}^{-1}$ we can observe two small vibrations caused by the resonance effect of aromatic compounds. The presence of the carbonyl functional group is confirmed since peaks are observed from 1850 to 1650 ${cm}^{-1}$ corresponding to the stretching of the C–O bond. The intense and well-defined peak between 1650 and 1600 ${cm}^{-1}$ indicates the presence of carboxylic acids, while the two peaks around 1615 and 1495 ${cm}^{-1}$ indicate the presence of double bounds for the vibration of the C–C bond, confirming the presence of aromatic compounds. Finally, peaks from 1500 to 600 ${cm}^{-1}$ correspond to the fingerprint area, which is specific and unique; the bands present from 1000 to 1300 ${cm}^{-1}$ are due to vibrations of the C–O bonds found in esters, carboxyls, ethers and hydroxyls.

Other studies on the phytochemicals from guava leaf extracts show similar spectra. Lok et al. [49] analyzed guava leaf extracts obtained with three different solvents, namely distilled water, ethanol and n-hexane, and they also observed the stretching bands of the C–H and O-H bonds; on the other hand, Nagpal et al. [50] and Lahlou et al. [51] also reported the vibration of the functional groups C=O, O–H, C–O and C–H.

### 2.4. UPLC-MS

The purified methanolic extract obtained by Soxhlet was selected for analysis by UPLC-MS given its outstanding reducing activity and yield of polyphenolic compounds, in comparison with the other two techniques evaluated. The tentative identification of the phytochemicals is presented in Table 3.

In the extract, 13 compounds were identified, among these were quercetin, considered the most active and powerful antioxidant of guava leaves [52]; catechin; kaempferol; avicularin; and guavinoside B and C, all of which exhibit different beneficial activities for the human health; e.g., anti-inflammatory, antimicrobial and antitumor activity [53,54,55,56,57,58,59,60,61]. Therefore, guava leaves are a rich source of polyphenolic compounds and a potential nutraceutical. On the other hand, there were also compounds that could not be identified, giving rise to new investigations for the identification, characterization and in vitro evaluation of the activity of these phytochemicals.

For additional information, see the Supplementary Materials.

**Table 3:** Tentative identification of phenolic compounds in *Psidium guajava* L. leaf extracts.

Extraction Method	No.	Tentative Identity	Tr (min)	*m*/*z* exp	*m*/*z* Calculated	Molecular Formula	Fragments	Reference
Soxhlet with methanol	1	Not identified	0.749	249.0305	248.034588	$C_{19}H_{5}O$	113.0318, 181.0322, 207.0358	
	2	Vescalagin	7.71	933.2043	933.07178	$C_{41}H_{26}O_{26}$	466.1265, 179.0356, 289.1466	[17]
	3	Catechin	9.587	289.147	289.079587	$C_{15}H_{14}O_{6}$	179.0359, 207.0354, 287.1305, 245.1503	[17]
	4	Not identified	18.88	603.1823	602.181312	$C_{40}H_{27}O_{6}$	179.0356, 207.0351, 235.9931, 257.02	
	5	Casuarinin/Casuarictin Isomer	19.637	935.2216	935.08743	$C_{41}H_{28}O_{26}$	467.1334, 145.9832, 385.2003, 478.1260	[17]
	6	Not identified	33.071	381.0783	380.076847	$C_{24}H_{13}O_{5}$	379.0626, 301.1106, 299.0956	
	7	Not identified	33.475	381.0775	380.076847	$C_{24}H_{13}O_{5}$	299.0951, 301.110, 302.1134, 379.0617, 271.098	
	8	Quercetin glucuronide	34.788	477.1651	477.075289	$C_{21}H_{18}O_{13}$	463.1837, 299.0954, 301.1106	[17]
	9	Reynoutrin	37.414	433.1703	433.08546	$C_{20}H_{18}O_{11}$	431.1533, 181.0318, 235.9926, 300.1021, 415.2863	[17]
	10	Guajaverin	40.242	433.1713	433.08546	$C_{20}H_{18}O_{11}$	431.1543, 300.1037, 301.1096, 391.9761	[17]
	11	Avicularin	40.798	433.1707	433.08546	$C_{20}H_{18}O_{11}$	431.1534, 300.1033, 302.1121	[17]
	12	Myrciaphenone B	48.222	481.1956	481.106589	$C_{21}H_{22}O_{13}$	479.1792, 417.1714, 365.9648, 257.0243, 235.9927, 239.9696, 207.0348, 181.0316, 179.0352	[17]
	13	Guavinoside C	57.06	585.1982	585.096419	$C_{27}H_{22}O_{15}$	583.1833, 304.9899, 285.9811, 235.9928, 257.0249, 352.9331	[17]
	14	Not identified	63.473	551.2103	550.207527	$C_{34}H_{31}O_{7}$	541.1789, 343.1274, 328.1021,	
	15	Guavinoside B	64.887	571.2532	571.15354	$C_{28}H_{28}O_{13}$	569.2360, 481.2711, 257.0239	[17]
	16	Not identified	78.22	711.5146	710.401989	$C_{30}H_{63}O_{18}$	701.4839, 549.4456, 503.4370	
	17	Not identified	82.967	695.5202	694.51974	$C_{51}H_{67}O$	685.4873, 533.4503, 487.4390	
	18	Luteolin 7-*O*-malonyl-glucoside	86.2	533.4523	533.101504	$C_{24}H_{22}O_{14}$	487.4415, 488.445, 523.4203, 501.4174	[62]
	19	Kaempferol 3-*O*-(6″-malonyl-glucoside)	86.907	533.4519	533.101504	$C_{24}H_{22}O_{14}$	487.4413, 488.4441, 523.4199	[62]
	20	Chrysoeriol 7-*O*-(6″-malonyl-glucoside)	87.816	547.4318	547.117154	$C_{25}H_{24}O_{14}$	501.4212, 502.4242, 427.0584, 533.4493	[62]

## 3. Materials and Methods

### 3.1. Plant Material

The collection of guava leaves was carried out manually and randomly from different specimens free of pesticides in Aguascalientes, Mexico, in November 2021. The plant sample was transferred in a botanical press to the laboratory where only green leaves, without damage from insects or pests, were selected. Subsequently, the leaves were thoroughly washed with distilled water to remove traces of dust and other contaminants and dried at 40 °C for 72 h [35,63]. Finally, the sample was pulverized with an electric processor and the obtained powder was stored at room temperature in an airtight container protected from light [64].

### 3.2. Extraction of Phytochemicals

Three different extraction techniques were tested: Soxhlet, maceration and ultrasound-assisted extraction (UAE). In all cases, a solid–liquid ratio of 1:20 was used (5 g of plant sample per 100 mL of solvent). It was decided that two different solvents should be used, namely methanol, since multiple articles [65,66,67] and previous work performed by our laboratory have demonstrated its better extractive power, and distilled water, as a green alternative. The continuous extraction by Soxhlet was carried out using 7 siphons [68], while the maceration lasted 8 days and 2 temperatures were evaluated: 25 °C and 37 °C [69,70,71]. The ultrasound-assisted extraction lasted 40 min and 2 temperatures were evaluated: 23 °C and 30 °C [72,73,74,75]. The extracts obtained via maceration and UAE were centrifuged (5000 rpm for 17 min) and filtered (0.2 µm) to eliminate guava leaf particles [17].

### 3.3. Solvent Elimination

The aqueous extracts were subjected to low temperatures (−48 °C) and a vacuum for 5 days in alyophilizer (FreeZone, Labconco, Kansas City, MO, USA) to remove distilled water [76]. On the other hand, the methanolic extracts were subjected to 50 °C in an oven to eliminate the solvent [32]. In all cases, a green to reddish brown powder was obtained, which was stored in an Eppendorf tube at room temperature protected from light until use.

### 3.4. Purification of Polyphenolic Compounds

Phenolic compounds from guava leaves were purified with the commercial adsorbent Amberlite XAD-16 (Sigma-Aldrich, Saint Louis, MO, USA). Briefly, the lyophilized aqueous extracts were solubilized in distilled water while the methanolic extracts were solubilized in 80% methanol and the alcohol was removed using a rotary evaporator to obtain the water solubilized extract. Subsequently, 20 mL of the extracts were added to a column packed with amberlite XAD-16 as a stationary phase and distilled water was added to eliminate sugars and other compounds present in the extract, and finally, the polyphenolic compounds were eluted with absolute ethanol. The solvent was removed in an oven at 50 °C for 24 h and the crystals obtained were kept protected from light at room temperature [32,77].

The yield of polyphenols per gram of plant material was determined as follows:

$$Yield=\frac{Milligrams\;of\;phenolic\;compounds\;obtained}{Grams\;of\;plant\;material\;used\;for\;extraction}$$

### 3.5. Antioxidant Capacity Tests

The antioxidant capacity of both the crude guava leaf extracts and their purified polyphenolic compounds was analyzed. Three different assays were carried out in microplates: Folin–Ciocalteu, ABTS and FRAP.

The reducing power was determined by adding 25 μL of Folin–Ciocalteu reagent and 25 μL of sodium carbonate (75 g/L) to 25 μL of properly diluted sample (1:4 *v*/*v*). The obtained mixture was homogenized and incubated at 40 °C for 30 min. After that, 200 μL of distilled H_2_O were added, and the absorbance at 750 nm was recorded [78]. Results were expressed as gallic acid equivalents in micrograms per milliliter (GAE μg/mL) using the calibration curve prepared with the same standard.

The ABTS^•+^ radical scavenging capacity assay was carried out according to the methodology proposed by Hernández et al. [77]. Briefly, a solution of ABTS (7 mM) and one of potassium persulfate (2.45 mM) were mixed (2:1) and were allowed to rest for 12 h at room temperature; then, it was adjusted with absolute ethanol until it reached an absorbance of 0.7 nm. Subsequently, five microliters of each test sample and calibration curve were pipetted in triplicate into the microplate, and 95 μL of the adjusted ABTS^•+^ solution was added. After 1 min the absorbance at 734 nm was measured. According to the Trolox calibration curve, results were expressed as the percentage inhibition of ABTS^•+^ radicals or as ${IC}_{50}$ (sample concentration needed to inhibit 50% of radicals).

The iron-reducing antioxidant power (FRAP) was determined by mixing 5 µL of the samples to be analyzed with 12 µL of phosphate buffer (pH 7) and 22 µL of 1% potassium ferrocyanide, and then incubated at 50 °C for 20 min. Then, 12 µL of 10% trichloroacetic acid, 45 µL of distilled water and 10 µL of ferric chloride were added to read the absorbance at 700 nm [79]. Results were reported as the μg gallic acid equivalent per milliliter (GAE μg/mL).

Initially, as a preliminary test, antioxidant capacity tests were carried out with solutions of the extracts (both crude and purified) at 0.250 mg·mL^−1^ to observe their behavior. Subsequently, the ${IC}_{50}$ of the purified extracts were determined with the ABTS^•+^ assay, and finally, the FRAP and Folin–Ciocalteu assays were performed with the purified extracts at their determined ${IC}_{50}$.

Statistical analysis was performed using Excel, MiniTab and GraphPad Prism 8.0.1 software.

### 3.6. FTIR (Fourier Transform Infrared Spectroscopy)

The polyphenolic compounds of the methanolic extracts obtained via Soxhlet, maceration at 25 °C and ultrasound at 30 °C were analyzed using Fourier transform infrared spectroscopy (Agilent Technologies, Santa Clara, CA, USA, FTIR model Cary 630 coupled to a zinc selenide crystal (ZnSe) ATR). The obtained powder after the purification process was deposited on the surface of the reader and secured by means of the equipped press. The spectra were acquired at a range of 4000–600 cm^−1^ across 32 scans with a resolution of 2 cm^−1^ [80].

### 3.7. UPLC-MS

The analysis was carried out with an Acquity UPLC system (Waters, Milford, MA, USA) consisting of an auto-sampler, a binary pump equipped with a 10 µL Loop (partial Loop injection mode) and a BEH PHENYL column (2.1 mm × 100 mm, 1.7 µm; WATERS, Waxford, Ireland). The solvents used were (A) water + 0.1% (*v*/*v*) formic acid and (B) acetonitrile + 0.1% (*v*/*v*) formic acid at a constant flow rate of 0.3 ${mL\cdot min}^{-1}$. The elution gradient (for 113 min) was 100% A, gradually decreasing until reaching 10% A and 90% B, to move from normal conditions (100% A) one minute later to re-equilibrate the column. MS detection was performed on a Q-ToF quadrupole orthogonal acceleration time-of-flight mass spectrometer (Q-TOF™, Waters, Milford, MA, USA) equipped with an electrospray ionization source (ESI). The sample acquisition mode was in negative ionic polarity, analysis mode in sensitivity and normal dynamic range, in a mass range of 50 to 1200 Da, sweep conditions of 0.5 $s^{-1}$, a centroid data format, a collision energy of 6 V and a cone voltage of 40 V.

## 4. Conclusions

The present study shows that guava leaves are a rich resource in polyphenolic compounds, the antioxidant activity of which could be of great interest to the pharmaceutical and food industry; thus, guava-producing countries should value this agro-industrial by-product.

About 13 compounds were identified in the analyzed guava leaf extract, including quercetin, catechin, hyperin and guajaverin. Of the three methods studied, Soxhlet is postulated as an advantageous option for the recovery of phenolic compounds, given that it does not require a significant amount of time, no processes are needed to separate the plant material from the solvent, the solvent can be reused and the extract obtained exhibits outstanding reducing activity and yield in phenolic compounds compared to maceration and ultrasound-assisted extraction. In addition, different commercially available systems allow the Soxhlet extraction method to be scaled and even automated for industrial applications. Furthermore, the purification of phenolic compounds generated improvements in the antioxidant activity in the three assays carried out (FRAP, Folin–Ciocalteu and ABTS^•+^) in our study, demonstrating that this process may be a way to increase the bioactivity of these phytochemicals.

It should be noted that, for its future industrial application, it will be necessary to face different challenges, especially to improve the long-term stability of polyphenolic compounds and increase their bioavailability; this can be achieved, for example, through encapsulation. In addition, more studies are needed on its toxicity, in vivo activity and safety.

## Figures and Tables

**Table 1:** Extraction yield of purified polyphenolic compounds.

Extraction Method				Extraction Yield
Technique	Solvent	Temperature (°C)	Time (min)	mg of Phenolic Compounds/g of Guava Leaf
Soxhlet	Methanol	65	5	44
Soxhlet	Distilled water	100	5	24
Maceration	Methanol	37	192	21
Maceration	Methanol	25	192	22
Ultrasound	Methanol	30	0.66	18
Ultrasound	Methanol	23	0.66	19
Ultrasound	Distilled water	30	0.66	45

**Table 2:** ${IC}_{50}$ values according to the extraction method obtained via the ABTS^•+^ assay.

Extraction Method	${IC}_{50}$ (µg·mL^−1^)
Soxhlet, methanol, 65 °C, 5 h	${119\pm 6}^{\;B}$
Soxhlet, distilled water, 100 °C, 5 h	${137\pm 12}^{\;AB}$
Maceration, methanol, 37 °C, 192 h	${89\pm 3}^{\;C}$
Maceration, methanol, 25 °C, 192 h	${87\pm 6}^{\;C}$
Ultrasound, methanol, 30 °C, 0.66 h	${78\pm 4}^{\;C}$
Ultrasound, methanol, 23 °C, 0.66 h	${78\pm 4}^{\;C}$
Ultrasound, distilled water, 30 °C, 0.66 h	${123\pm 4}^{\;B}$
Ultrasound, distilled water, 23 °C, 0.66 h	${152\pm 12}^{\;A}$

Different letters mean significant (*p* < 0.05) differences between the extraction methods.

## Data Availability

Data are contained within the article and Supplementary Materials.

## Supplementary Materials

The following supporting information can be downloaded at: https://www.mdpi.com/article/10.3390/molecules29010085/s1.
