# Supplementary material for: Influence of the Extraction Method on the Polyphenolic Profile and the Antioxidant Activity of Psidium guajava L. Leaf Extracts
Source: Molecules. 2023 Dec 22;29(1):85. doi: 10.3390/molecules29010085 (PMC10779645; doi:10.3390/molecules29010085)
Supplement: Supplementary file 1 [file molecules-29-00085-s001.zip › molecules-2744695-supplementary.pdf]

Supplementary material 1: Raw data UPLC-MS Soxhlet methanol

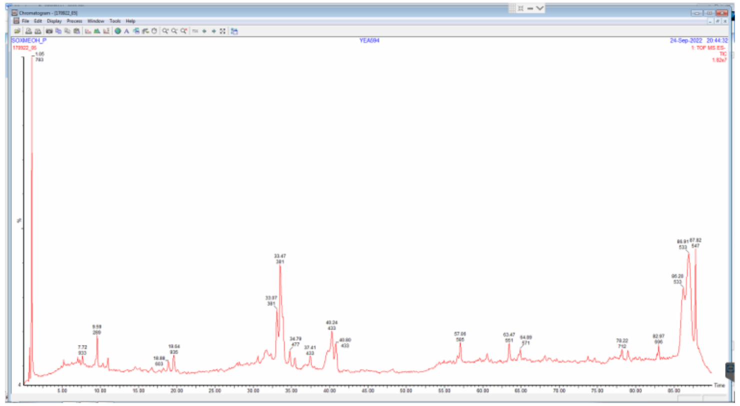

molecules

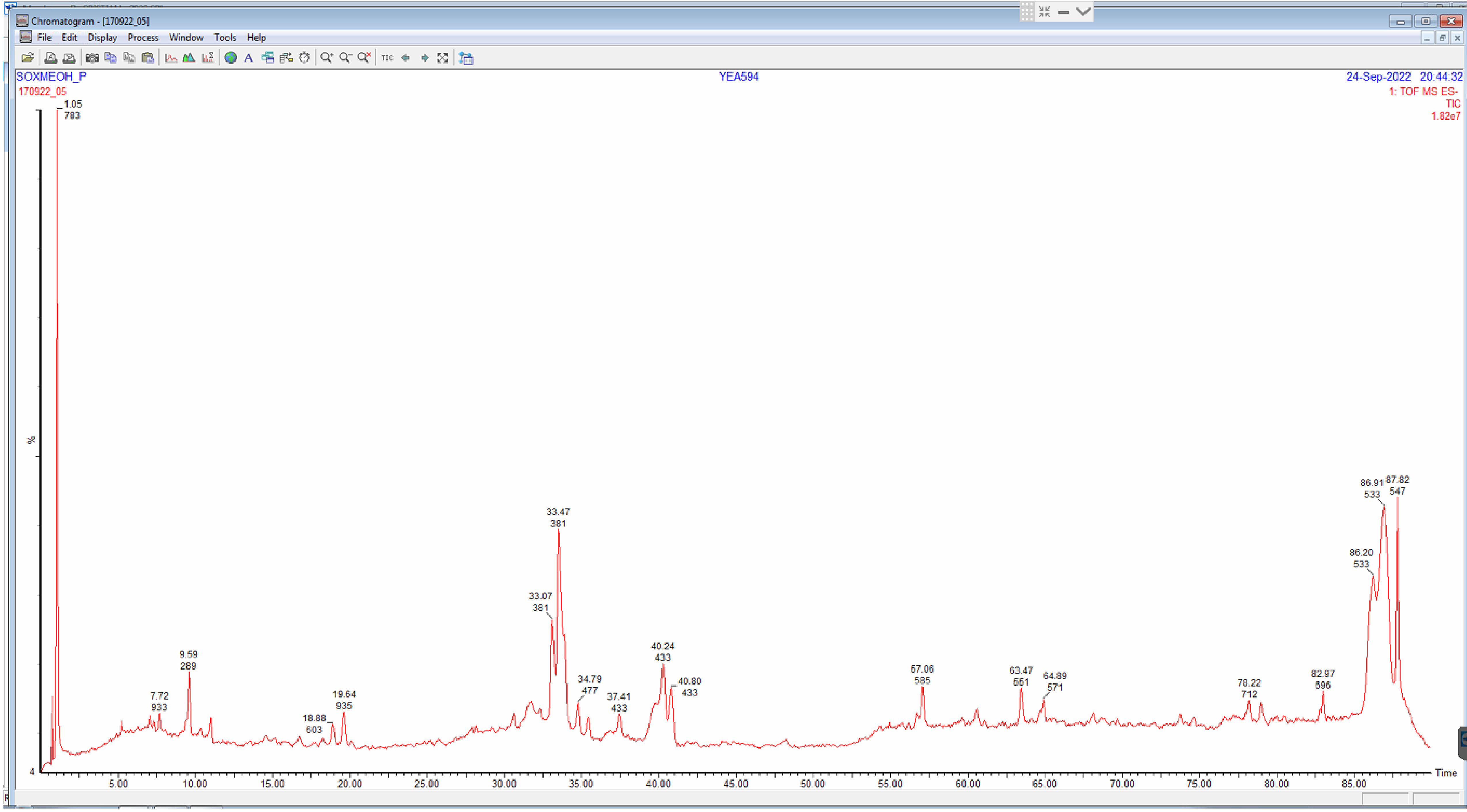

Chromatogram - [170922_05]
File Edit Display Process Window Tools Help
SOXMEOH_P
170922_05
YEA594
24-Sep-2022 20:44:32
1: TOF MS ES-
TIC
1.82e7
%
1.05
783
7.72
933
9.59
289
18.88
603
19.64
935
33.07
381
33.47
381
34.79
477
37.41
433
40.24
433
40.80
433
57.06
585
63.47
551
64.89
571
78.22
712
82.97
696
86.20
533
86.91
533
87.82
547
4
5.00 10.00 15.00 20.00 25.00 30.00 35.00 40.00 45.00 50.00 55.00 60.00 65.00 70.00 75.00 80.00 85.00
Time

Spectrum - [170922_05]

File Edit Display Process Tools Window Help

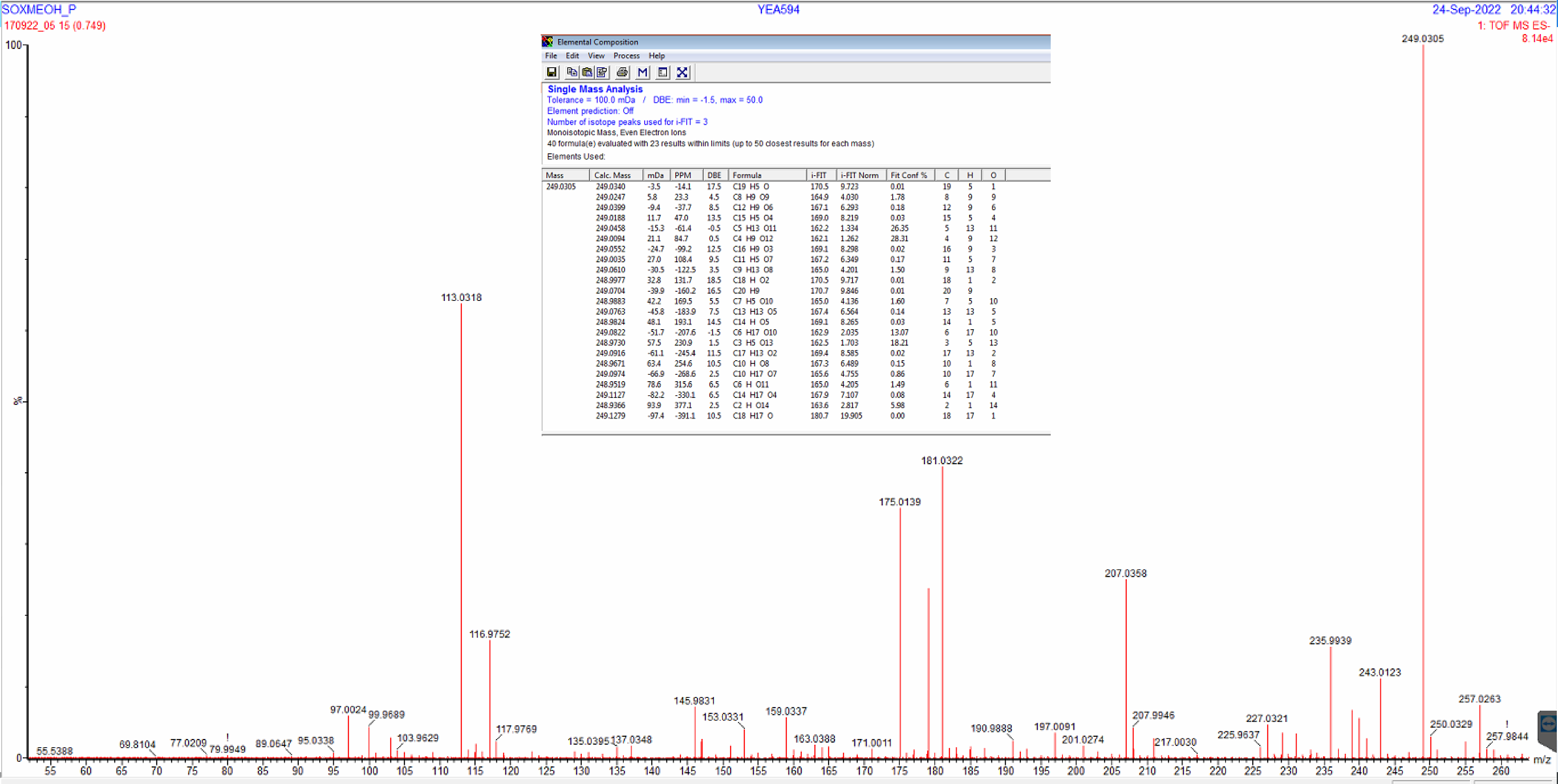

Elemental Composition

File Edit View Process Help

Single Mass Analysis

Tolerance = 100.0 mDa / DBE: min = -1.5, max = 50.0

Element prediction: Off

Number of isotope peaks used for i-FIT = 3

Monoisotopic Mass, Even Electron Ions

40 formula(e) evaluated with 23 results within limits (up to 50 closest results for each mass)

Elements Used:

Mass	Calc. Mass	mDa	PPM	DBE	Formula	i-FIT	i-FIT Norm	Fit Conf %	C	H	O
249.0305	249.0340	-3.5	-14.1	17.5	C19 H5 O	170.5	9.723	0.01	19	5	1
	249.0247	5.8	23.3	4.5	C8 H9 O9	164.9	4.030	1.78	8	9	9
	249.0399	-9.4	-37.7	8.5	C12 H9 O6	167.1	6.293	0.18	12	9	6
	249.0188	11.7	47.0	13.5	C15 H5 O4	169.0	8.219	0.03	15	5	4
	249.0458	-15.3	-61.4	-0.5	C5 H13 O11	162.2	1.334	26.35	5	13	11
	249.0094	21.1	84.7	0.5	C4 H9 O12	162.1	1.262	28.31	4	9	12
	249.0552	-24.7	-99.2	12.5	C16 H9 O3	169.1	8.298	0.02	16	9	3
	249.0035	27.0	108.4	9.5	C11 H5 O7	167.2	6.349	0.17	11	5	7
	249.0610	-30.5	-122.5	3.5	C9 H13 O8	165.0	4.201	1.50	9	13	8
	248.9977	32.8	131.7	18.5	C18 H O2	170.5	9.717	0.01	18	1	2
	249.0704	-39.9	-160.2	16.5	C20 H9	170.7	9.846	0.01	20	9	
	248.9883	42.2	169.5	5.5	C7 H5 O10	165.0	4.136	1.60	7	5	10
	249.0763	-45.8	-183.9	7.5	C13 H13 O5	167.4	6.564	0.14	13	13	5
	248.9824	48.1	193.1	14.5	C14 H O5	169.1	8.265	0.03	14	1	5
	249.0822	-51.7	-207.6	-1.5	C6 H17 O10	162.9	2.035	13.07	6	17	10
	248.9730	57.5	230.9	1.5	C3 H5 O13	162.5	1.703	18.21	3	5	13
	249.0916	-61.1	-245.4	11.5	C17 H13 O2	169.4	8.585	0.02	17	13	2
	248.9671	63.4	254.6	10.5	C10 H O8	167.3	6.489	0.15	10	1	8
	249.0974	-66.9	-268.6	2.5	C10 H17 O7	165.6	4.755	0.86	10	17	7
	248.9519	78.6	315.6	6.5	C6 H O11	165.0	4.205	1.49	6	1	11
	249.1127	-82.2	-330.1	6.5	C14 H17 O4	167.9	7.107	0.08	14	17	4
	248.9366	93.9	377.1	2.5	C2 H O14	163.6	2.817	5.98	2	1	14
	249.1279	-97.4	-391.1	10.5	C18 H17 O	180.7	19.905	0.00	18	17	1

Spectrum - [170922_05]

File Edit Display Process Tools Window Help

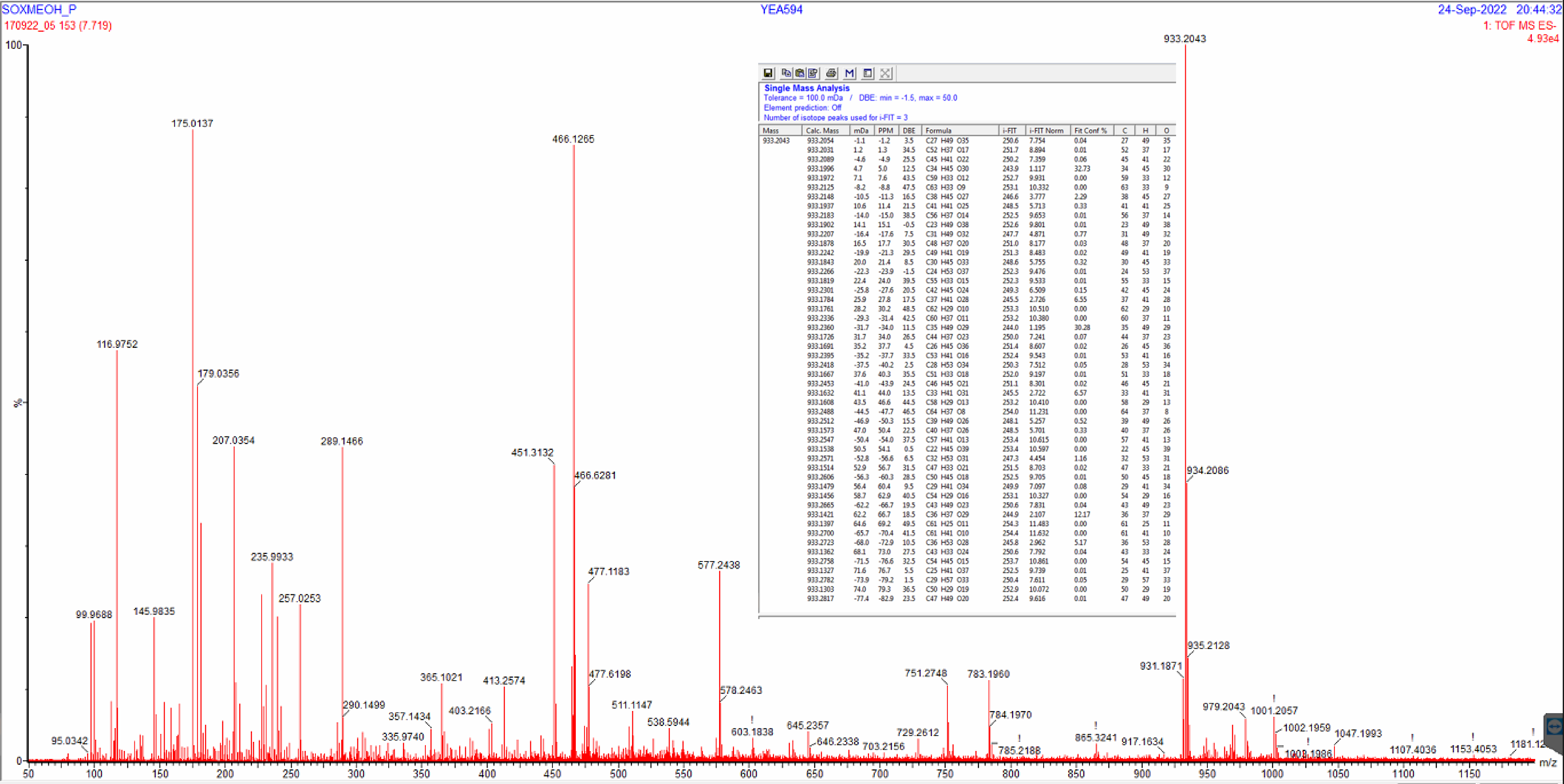

Single Mass Analysis

Tolerance = 100.0 mDa / DBE: min = -1.5, max = 50.0

Element prediction: Off

Number of isotope peaks used for i-FIT = 3

Mass	Calc. Mass	mDa	PPM	DBE	Formula	i-FIT	i-FIT Norm	Fit Conf %	C	H	O
933.2043	933.2054	-1.1	-1.2	3.5	C27 H49 O35	250.6	7.754	0.04	27	49	35
	933.2031	1.2	1.3	34.5	C52 H37 O17	251.7	8.894	0.01	52	37	17
	933.2089	-4.6	-4.9	25.5	C45 H41 O22	250.2	7.359	0.06	45	41	22
	933.1996	4.7	5.0	12.5	C34 H45 O30	243.9	1.117	32.73	34	45	30
	933.1972	7.1	7.6	43.5	C59 H33 O12	252.7	9.931	0.00	59	33	12
	933.2125	-8.2	-8.8	47.5	C63 H33 O9	253.1	10.332	0.00	63	33	9
	933.2148	-10.5	-11.3	16.5	C38 H45 O27	246.6	3.777	2.29	38	45	27
	933.1937	10.6	11.4	21.5	C41 H41 O25	248.5	5.713	0.33	41	41	25
	933.2183	-14.0	-15.0	38.5	C56 H37 O14	252.5	9.653	0.01	56	37	14
	933.1902	14.1	15.1	-0.5	C23 H49 O38	252.6	9.801	0.01	23	49	38
	933.2207	-16.4	-17.6	7.5	C31 H49 O32	247.7	4.871	0.77	31	49	32
	933.1878	16.5	17.7	30.5	C48 H37 O20	251.0	8.177	0.03	48	37	20
	933.2242	-19.9	-21.3	29.5	C49 H41 O19	251.3	8.483	0.02	49	41	19
	933.1843	20.0	21.4	8.5	C30 H45 O33	248.6	5.755	0.32	30	45	33
	933.2266	-22.3	-23.9	-1.5	C24 H53 O37	252.3	9.476	0.01	24	53	37
	933.1819	22.4	24.0	39.5	C55 H33 O15	252.3	9.533	0.01	55	33	15
	933.2301	-25.8	-27.6	20.5	C42 H45 O24	249.3	6.509	0.15	42	45	24
	933.1784	25.9	27.8	17.5	C37 H41 O28	245.5	2.726	6.55	37	41	28
	933.1761	28.2	30.2	48.5	C62 H29 O10	253.3	10.510	0.00	62	29	10
	933.2336	-29.3	-31.4	42.5	C60 H37 O11	253.2	10.380	0.00	60	37	11
	933.2360	-31.7	-34.0	11.5	C35 H49 O29	244.0	1.195	30.28	35	49	29
	933.1726	31.7	34.0	26.5	C44 H37 O23	250.0	7.241	0.07	44	37	23
	933.1691	35.2	37.7	4.5	C26 H45 O36	251.4	8.607	0.02	26	45	36
	933.2395	-35.2	-37.7	33.5	C53 H41 O16	252.4	9.543	0.01	53	41	16
	933.2418	-37.5	-40.2	2.5	C28 H53 O34	250.3	7.512	0.05	28	53	34
	933.1667	37.6	40.3	35.5	C51 H33 O18	252.0	9.197	0.01	51	33	18
	933.2453	-41.0	-43.9	24.5	C46 H45 O21	251.1	8.301	0.02	46	45	21
	933.1632	41.1	44.0	13.5	C33 H41 O31	245.5	2.722	6.57	33	41	31
	933.1608	43.5	46.6	44.5	C58 H29 O13	253.2	10.410	0.00	58	29	13
	933.2488	-44.5	-47.7	46.5	C64 H37 O8	254.0	11.231	0.00	64	37	8
	933.2512	-46.9	-50.3	15.5	C39 H49 O26	248.1	5.257	0.52	39	49	26
	933.1573	47.0	50.4	22.5	C40 H37 O26	248.5	5.701	0.33	40	37	26
	933.2547	-50.4	-54.0	37.5	C57 H41 O13	253.4	10.615	0.00	57	41	13
	933.1538	50.5	54.1	0.5	C22 H45 O39	253.4	10.597	0.00	22	45	39
	933.2571	-52.8	-56.6	6.5	C32 H53 O31	247.3	4.454	1.16	32	53	31
	933.1514	52.9	56.7	31.5	C47 H33 O21	251.5	8.703	0.02	47	33	21
	933.2606	-56.3	-60.3	28.5	C50 H45 O18	252.5	9.705	0.01	50	45	18
	933.1479	56.4	60.4	9.5	C29 H41 O34	249.9	7.097	0.08	29	41	34
	933.1456	58.7	62.9	40.5	C54 H29 O16	253.1	10.327	0.00	54	29	16
	933.2665	-62.2	-66.7	19.5	C43 H49 O23	250.6	7.831	0.04	43	49	23
	933.1421	62.2	66.7	18.5	C36 H37 O29	244.9	2.107	12.17	36	37	29
	933.1397	64.6	69.2	49.5	C61 H25 O11	254.3	11.483	0.00	61	25	11
	933.2700	-65.7	-70.4	41.5	C61 H41 O10	254.4	11.632	0.00	61	41	10
	933.2723	-68.0	-72.9	10.5	C36 H53 O28	245.8	2.962	5.17	36	53	28
	933.1362	68.1	73.0	27.5	C43 H33 O24	250.6	7.792	0.04	43	33	24
	933.2758	-71.5	-76.6	32.5	C54 H45 O15	253.7	10.861	0.00	54	45	15
	933.1327	71.6	76.7	5.5	C25 H41 O37	252.5	9.739	0.01	25	41	37
	933.2782	-73.9	-79.2	1.5	C29 H57 O33	250.4	7.611	0.05	29	57	33
	933.1303	74.0	79.3	36.5	C50 H29 O19	252.9	10.072	0.00	50	29	19
	933.2817	-77.4	-82.9	23.5	C47 H49 O20	252.4	9.616	0.01	47	49	20

Desktop 13:55

Spectrum - [170922_05]

File Edit Display Process Tools Window Help

SOXMEOH_P YEA594 24-Sep-2022 20:44:32

170922_05 190 (9.587) 1: TOF MS ES- 6.85e5

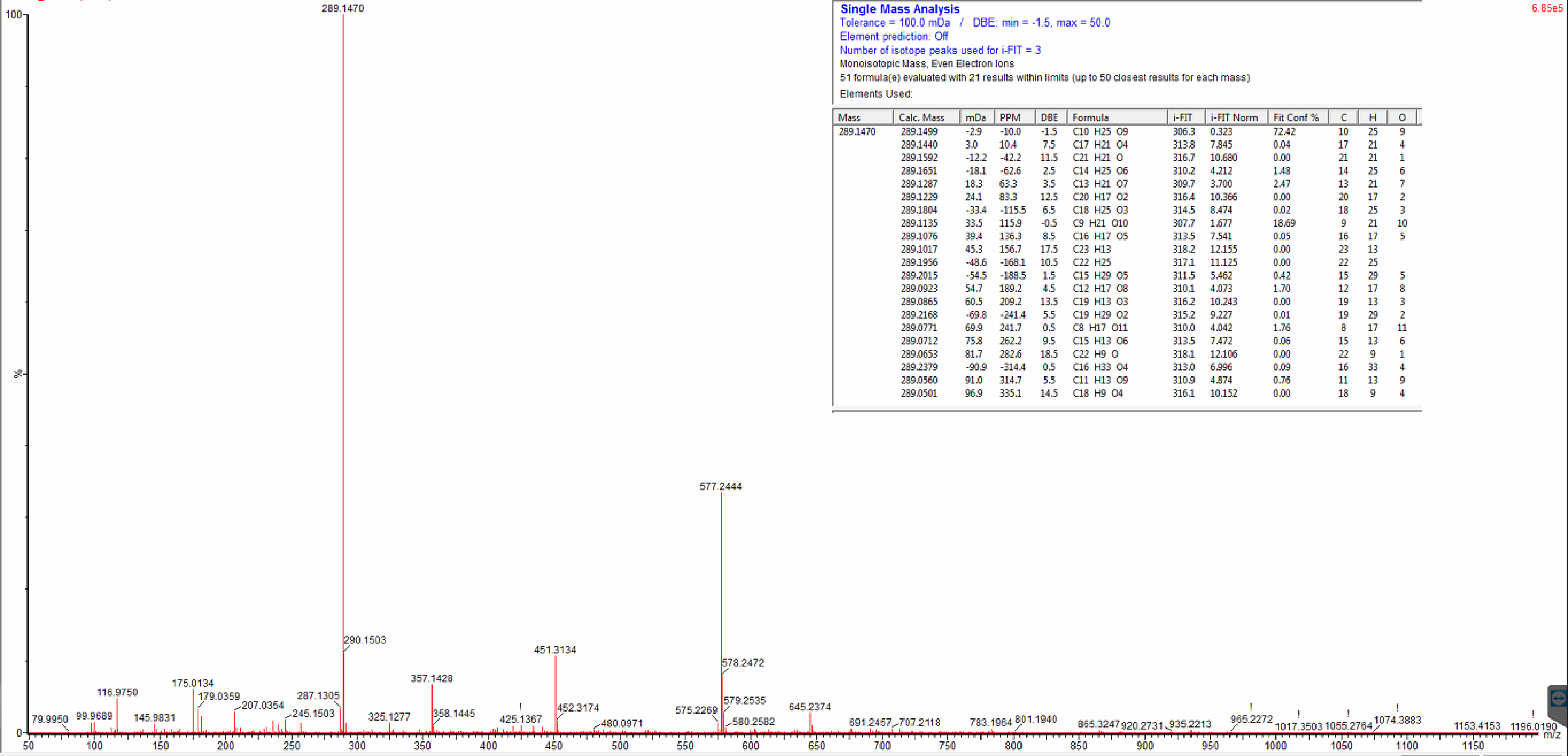

Single Mass Analysis

Tolerance = 100.0 mDa / DBE: min = -1.5, max = 50.0

Element prediction: Off

Number of isotope peaks used for i-FIT = 3

Monoisotopic Mass, Even Electron Ions

51 formula(e) evaluated with 21 results within limits (up to 50 closest results for each mass)

Elements Used:

Mass	Calc. Mass	mDa	PPM	DBE	Formula	i-FIT	i-FIT Norm	Fit Conf %	C	H	O
289.1470	289.1499	-2.9	-10.0	-1.5	C10 H25 O9	306.3	0.323	72.42	10	25	9
	289.1440	3.0	10.4	7.5	C17 H21 O4	313.8	7.845	0.04	17	21	4
	289.1592	-12.2	-42.2	11.5	C21 H21 O	316.7	10.680	0.00	21	21	1
	289.1651	-18.1	-62.6	2.5	C14 H25 O6	310.2	4.212	1.48	14	25	6
	289.1287	18.3	63.3	3.5	C13 H21 O7	309.7	3.700	2.47	13	21	7
	289.1229	24.1	83.3	12.5	C20 H17 O2	316.4	10.366	0.00	20	17	2
	289.1804	-33.4	-115.5	6.5	C18 H25 O3	314.5	8.474	0.02	18	25	3
	289.1135	33.5	115.9	-0.5	C9 H21 O10	307.7	1.677	18.69	9	21	10
	289.1076	39.4	136.3	8.5	C16 H17 O5	313.5	7.541	0.05	16	17	5
	289.1017	45.3	156.7	17.5	C23 H13	318.2	12.155	0.00	23	13	
	289.1956	-48.6	-168.1	10.5	C22 H25	317.1	11.125	0.00	22	25	
	289.2015	-54.5	-188.5	1.5	C15 H29 O5	311.5	5.462	0.42	15	29	5
	289.0923	54.7	189.2	4.5	C12 H17 O8	310.1	4.073	1.70	12	17	8
	289.0865	60.5	209.2	13.5	C19 H13 O3	316.2	10.243	0.00	19	13	3
	289.2168	-69.8	-241.4	5.5	C19 H29 O2	315.2	9.227	0.01	19	29	2
	289.0771	69.9	241.7	0.5	C8 H17 O11	310.0	4.042	1.76	8	17	11
	289.0712	75.8	262.2	9.5	C15 H13 O6	313.5	7.472	0.06	15	13	6
	289.0653	81.7	282.6	18.5	C22 H9 O	318.1	12.106	0.00	22	9	1
	289.2379	-90.9	-314.4	0.5	C16 H33 O4	313.0	6.996	0.09	16	33	4
	289.0560	91.0	314.7	5.5	C11 H13 O9	310.9	4.874	0.76	11	13	9
	289.0501	96.9	335.1	14.5	C18 H9 O4	316.1	10.152	0.00	18	9	4

Desktop 13:58

Spectrum - [170922_05]

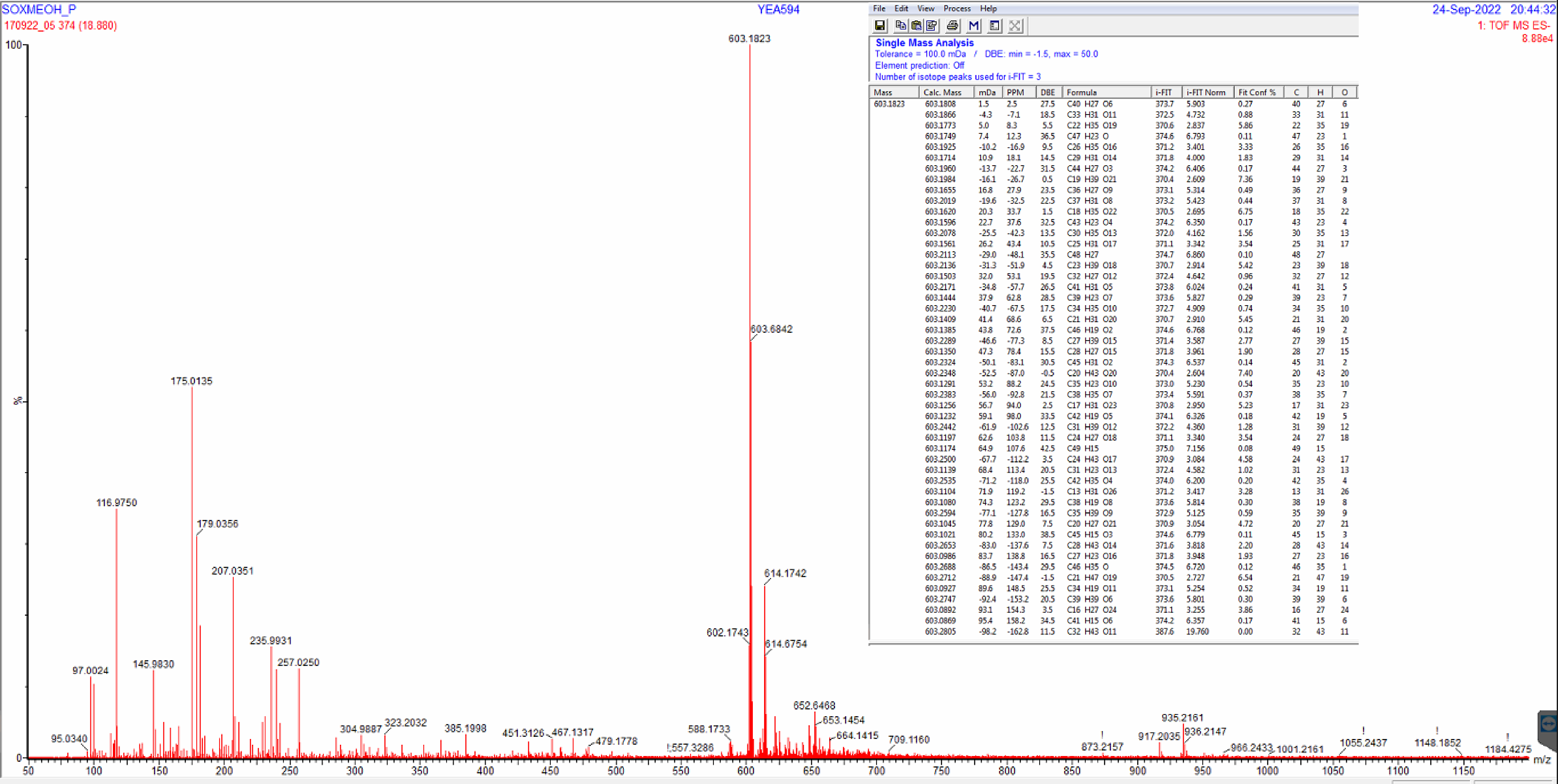

Single Mass Analysis

Tolerance = 100.0 mDa / DBE: min = -1.5, max = 50.0

Element prediction: Off

Number of isotope peaks used for i-FIT = 3

Mass	Calc. Mass	mDa	PPM	DBE	Formula	i-FIT	i-FIT Norm	Fit Conf %	C	H	O
603.1823	603.1808	1.5	2.5	27.5	C40 H27 O6	373.7	5.903	0.27	40	27	6
	603.1866	-4.3	-7.1	18.5	C33 H31 O11	372.5	4.732	0.88	33	31	11
	603.1773	5.0	8.3	5.5	C22 H35 O19	370.6	2.837	5.86	22	35	19
	603.1749	7.4	12.3	36.5	C47 H23 O	374.6	6.793	0.11	47	23	1
	603.1925	-10.2	-16.9	9.5	C26 H35 O16	371.2	3.401	3.33	26	35	16
	603.1714	10.9	18.1	14.5	C29 H31 O14	371.8	4.000	1.83	29	31	14
	603.1960	-13.7	-22.7	31.5	C44 H27 O3	374.2	6.406	0.17	44	27	3
	603.1984	-16.1	-26.7	0.5	C19 H39 O21	370.4	2.609	7.36	19	39	21
	603.1655	16.8	27.9	23.5	C36 H27 O9	373.1	5.314	0.49	36	27	9
	603.2019	-19.6	-32.5	22.5	C37 H31 O8	373.2	5.423	0.44	37	31	8
	603.1620	20.3	33.7	1.5	C18 H35 O22	370.5	2.695	6.75	18	35	22
	603.1596	22.7	37.6	32.5	C43 H23 O4	374.2	6.350	0.17	43	23	4
	603.2078	-25.5	-42.3	13.5	C30 H35 O13	372.0	4.162	1.56	30	35	13
	603.1561	26.2	43.4	10.5	C25 H31 O17	371.1	3.342	3.54	25	31	17
	603.2113	-29.0	-48.1	35.5	C48 H27	374.7	6.860	0.10	48	27	
	603.2136	-31.3	-51.9	4.5	C23 H39 O18	370.7	2.914	5.42	23	39	18
	603.1503	32.0	53.1	19.5	C32 H27 O12	372.4	4.642	0.96	32	27	12
	603.2171	-34.8	-57.7	26.5	C41 H31 O5	373.8	6.024	0.24	41	31	5
	603.1444	37.9	62.8	28.5	C39 H23 O7	373.6	5.827	0.29	39	23	7
	603.2230	-40.7	-67.5	17.5	C34 H35 O10	372.7	4.909	0.74	34	35	10
	603.1409	41.4	68.6	6.5	C21 H31 O20	370.7	2.910	5.45	21	31	20
	603.1385	43.8	72.6	37.5	C46 H19 O2	374.6	6.768	0.12	46	19	2
	603.2289	-46.6	-77.3	8.5	C27 H39 O15	371.4	3.587	2.77	27	39	15
	603.1350	47.3	78.4	15.5	C28 H27 O15	371.8	3.961	1.90	28	27	15
	603.2324	-50.1	-83.1	30.5	C45 H31 O2	374.3	6.537	0.14	45	31	2
	603.2348	-52.5	-87.0	-0.5	C20 H43 O20	370.4	2.604	7.40	20	43	20
	603.1291	53.2	88.2	24.5	C35 H23 O10	373.0	5.230	0.54	35	23	10
	603.2383	-56.0	-92.8	21.5	C38 H35 O7	373.4	5.591	0.37	38	35	7
	603.1256	56.7	94.0	2.5	C17 H31 O23	370.8	2.950	5.23	17	31	23
	603.1232	59.1	98.0	33.5	C42 H19 O5	374.1	6.326	0.18	42	19	5
	603.2442	-61.9	-102.6	12.5	C31 H39 O12	372.2	4.360	1.28	31	39	12
	603.1197	62.6	103.8	11.5	C24 H27 O18	371.1	3.340	3.54	24	27	18
	603.1174	64.9	107.6	42.5	C49 H15	375.0	7.156	0.08	49	15	
	603.2500	-67.7	-112.2	3.5	C24 H43 O17	370.9	3.084	4.58	24	43	17
	603.1139	68.4	113.4	20.5	C31 H23 O13	372.4	4.582	1.02	31	23	13
	603.2535	-71.2	-118.0	25.5	C42 H35 O4	374.0	6.200	0.20	42	35	4
	603.1104	71.9	119.2	-1.5	C13 H31 O26	371.2	3.417	3.28	13	31	26
	603.1080	74.3	123.2	29.5	C38 H19 O8	373.6	5.814	0.30	38	19	8
	603.2594	-77.1	-127.8	16.5	C35 H39 O9	372.9	5.125	0.59	35	39	9
	603.1045	77.8	129.0	7.5	C20 H27 O21	370.9	3.054	4.72	20	27	21
	603.1021	80.2	133.0	38.5	C45 H15 O3	374.6	6.779	0.11	45	15	3
	603.2653	-83.0	-137.6	7.5	C28 H43 O14	371.6	3.818	2.20	28	43	14
	603.0986	83.7	138.8	16.5	C27 H23 O16	371.8	3.948	1.93	27	23	16
	603.2688	-86.5	-143.4	29.5	C46 H35 O	374.5	6.720	0.12	46	35	1
	603.2712	-88.9	-147.4	-1.5	C21 H47 O19	370.5	2.727	6.54	21	47	19
	603.0927	89.6	148.5	25.5	C34 H19 O11	373.1	5.254	0.52	34	19	11
	603.2747	-92.4	-153.2	20.5	C39 H39 O6	373.6	5.801	0.30	39	39	6
	603.0892	93.1	154.3	3.5	C16 H27 O24	371.1	3.255	3.86	16	27	24
	603.0869	95.4	158.2	34.5	C41 H15 O6	374.2	6.357	0.17	41	15	6
	603.2805	-98.2	-162.8	11.5	C32 H43 O11	387.6	19.760	0.00	32	43	11

Spectrum - [170922_05]

File Edit Display Process Tools Window Help

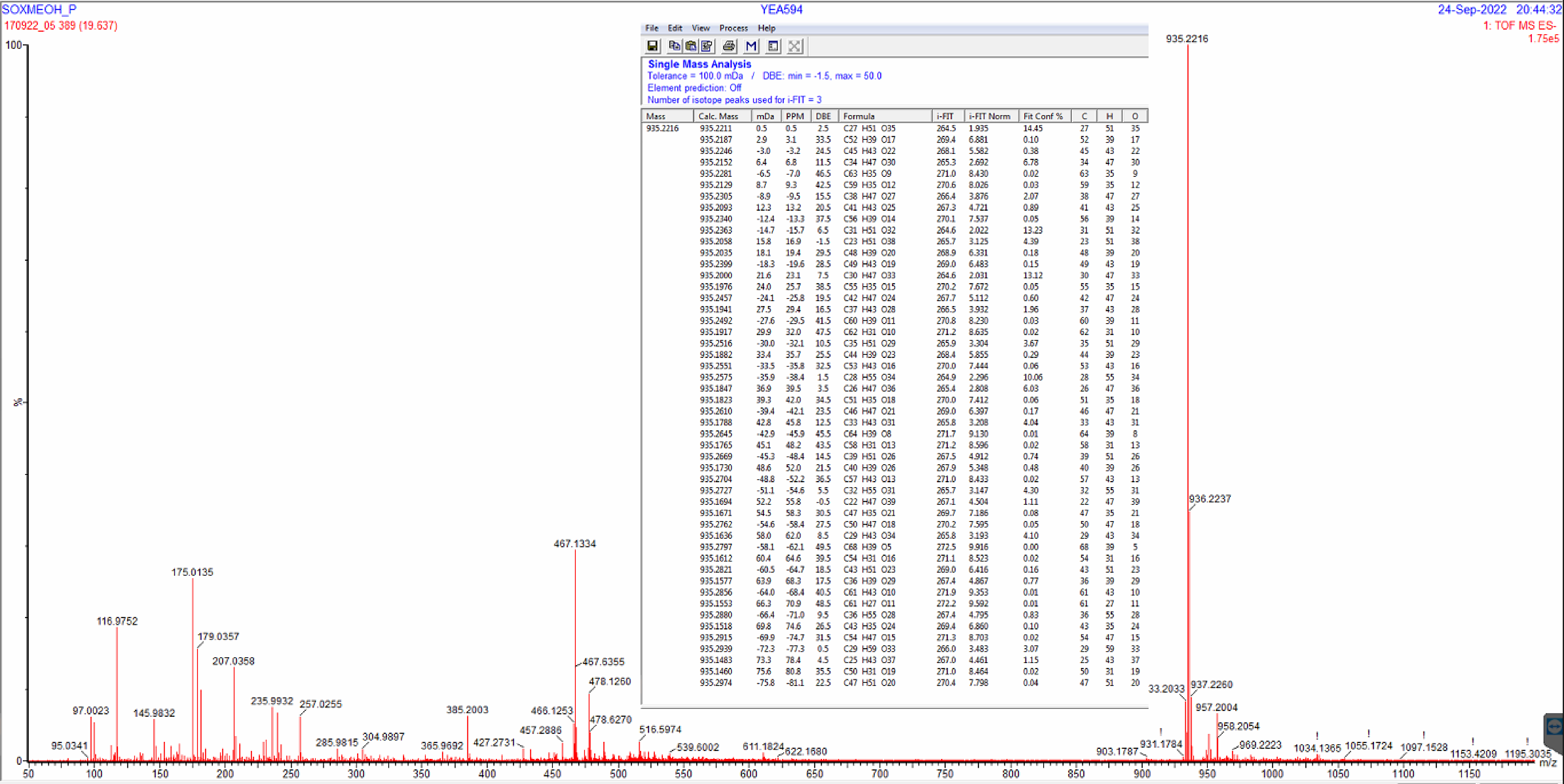

File Edit View Process Help

Single Mass Analysis

Tolerance = 100.0 mDa / DBE: min = -1.5, max = 50.0

Element prediction: Off

Number of isotope peaks used for i-FIT = 3

Mass	Calc. Mass	mDa	PPM	DBE	Formula	i-FIT	i-FIT Norm	Fit Conf %	C	H	O
935.2216	935.2211	0.5	0.5	2.5	$C_{27}H_{51}O_{35}$	264.5	1.935	14.45	27	51	35
	935.2187	2.9	3.1	33.5	$C_{52}H_{39}O_{17}$	269.4	6.881	0.10	52	39	17
	935.2246	-3.0	-3.2	24.5	$C_{45}H_{43}O_{22}$	268.1	5.582	0.38	45	43	22
	935.2152	6.4	6.8	11.5	$C_{34}H_{47}O_{30}$	265.3	2.692	6.78	34	47	30
	935.2281	-6.5	-7.0	46.5	$C_{63}H_{35}O_{9}$	271.0	8.430	0.02	63	35	9
	935.2129	8.7	9.3	42.5	$C_{59}H_{35}O_{12}$	270.6	8.026	0.03	59	35	12
	935.2305	-8.9	-9.5	15.5	$C_{38}H_{47}O_{27}$	266.4	3.876	2.07	38	47	27
	935.2093	12.3	13.2	20.5	$C_{41}H_{43}O_{25}$	267.3	4.721	0.89	41	43	25
	935.2340	-12.4	-13.3	37.5	$C_{56}H_{39}O_{14}$	270.1	7.537	0.05	56	39	14
	935.2363	-14.7	-15.7	6.5	$C_{31}H_{51}O_{32}$	264.6	2.022	13.23	31	51	32
	935.2058	15.8	16.9	-1.5	$C_{23}H_{51}O_{38}$	265.7	3.125	4.39	23	51	38
	935.2035	18.1	19.4	29.5	$C_{48}H_{39}O_{20}$	268.9	6.331	0.18	48	39	20
	935.2399	-18.3	-19.6	28.5	$C_{49}H_{43}O_{19}$	269.0	6.483	0.15	49	43	19
	935.2000	21.6	23.1	7.5	$C_{30}H_{47}O_{33}$	264.6	2.031	13.12	30	47	33
	935.1976	24.0	25.7	38.5	$C_{55}H_{35}O_{15}$	270.2	7.672	0.05	55	35	15
	935.2457	-24.1	-25.8	19.5	$C_{42}H_{47}O_{24}$	267.7	5.112	0.60	42	47	24
	935.1941	27.5	29.4	16.5	$C_{37}H_{43}O_{28}$	266.5	3.932	1.96	37	43	28
	935.2492	-27.6	-29.5	41.5	$C_{60}H_{39}O_{11}$	270.8	8.230	0.03	60	39	11
	935.1917	29.9	32.0	47.5	$C_{62}H_{31}O_{10}$	271.2	8.635	0.02	62	31	10
	935.2516	-30.0	-32.1	10.5	$C_{35}H_{51}O_{29}$	265.9	3.304	3.67	35	51	29
	935.1882	33.4	35.7	25.5	$C_{44}H_{39}O_{23}$	268.4	5.855	0.29	44	39	23
	935.2551	-33.5	-35.8	32.5	$C_{53}H_{43}O_{16}$	270.0	7.444	0.06	53	43	16
	935.2575	-35.9	-38.4	1.5	$C_{28}H_{55}O_{34}$	264.9	2.296	10.06	28	55	34
	935.1847	36.9	39.5	3.5	$C_{26}H_{47}O_{36}$	265.4	2.808	6.03	26	47	36
	935.1823	39.3	42.0	34.5	$C_{51}H_{35}O_{18}$	270.0	7.412	0.06	51	35	18
	935.2610	-39.4	-42.1	23.5	$C_{46}H_{47}O_{21}$	269.0	6.397	0.17	46	47	21
	935.1788	42.8	45.8	12.5	$C_{33}H_{43}O_{31}$	265.8	3.208	4.04	33	43	31
	935.2645	-42.9	-45.9	45.5	$C_{64}H_{39}O_{8}$	271.7	9.130	0.01	64	39	8
	935.1765	45.1	48.2	43.5	$C_{58}H_{31}O_{13}$	271.2	8.596	0.02	58	31	13
	935.2669	-45.3	-48.4	14.5	$C_{39}H_{51}O_{26}$	267.5	4.912	0.74	39	51	26
	935.1730	48.6	52.0	21.5	$C_{40}H_{39}O_{26}$	267.9	5.348	0.48	40	39	26
	935.2704	-48.8	-52.2	36.5	$C_{57}H_{43}O_{13}$	271.0	8.433	0.02	57	43	13
	935.2727	-51.1	-54.6	5.5	$C_{32}H_{55}O_{31}$	265.7	3.147	4.30	32	55	31
	935.1694	52.2	55.8	-0.5	$C_{22}H_{47}O_{39}$	267.1	4.504	1.11	22	47	39
	935.1671	54.5	58.3	30.5	$C_{47}H_{35}O_{21}$	269.7	7.186	0.08	47	35	21
	935.2762	-54.6	-58.4	27.5	$C_{50}H_{47}O_{18}$	270.2	7.595	0.05	50	47	18
	935.1636	58.0	62.0	8.5	$C_{29}H_{43}O_{34}$	265.8	3.193	4.10	29	43	34
	935.2797	-58.1	-62.1	49.5	$C_{68}H_{39}O_{5}$	272.5	9.916	0.00	68	39	5
	935.1612	60.4	64.6	39.5	$C_{54}H_{31}O_{16}$	271.1	8.523	0.02	54	31	16
	935.2821	-60.5	-64.7	18.5	$C_{43}H_{51}O_{23}$	269.0	6.416	0.16	43	51	23
	935.1577	63.9	68.3	17.5	$C_{36}H_{39}O_{29}$	267.4	4.867	0.77	36	39	29
	935.2856	-64.0	-68.4	40.5	$C_{61}H_{43}O_{10}$	271.9	9.353	0.01	61	43	10
	935.1553	66.3	70.9	48.5	$C_{61}H_{27}O_{11}$	272.2	9.592	0.01	61	27	11
	935.2880	-66.4	-71.0	9.5	$C_{36}H_{55}O_{28}$	267.4	4.795	0.83	36	55	28
	935.1518	69.8	74.6	26.5	$C_{43}H_{35}O_{24}$	269.4	6.860	0.10	43	35	24
	935.2915	-69.9	-74.7	31.5	$C_{54}H_{47}O_{15}$	271.3	8.703	0.02	54	47	15
	935.2939	-72.3	-77.3	0.5	$C_{29}H_{59}O_{33}$	266.0	3.483	3.07	29	59	33
	935.1483	73.3	78.4	4.5	$C_{25}H_{43}O_{37}$	267.0	4.461	1.15	25	43	37
	935.1460	75.6	80.8	35.5	$C_{50}H_{31}O_{19}$	271.0	8.464	0.02	50	31	19
	935.2974	-75.8	-81.1	22.5	$C_{47}H_{51}O_{20}$	270.4	7.798	0.04	47	51	20

Desktop 14:11

Spectrum - [170922_05]

File Edit Display Process Tools Window Help

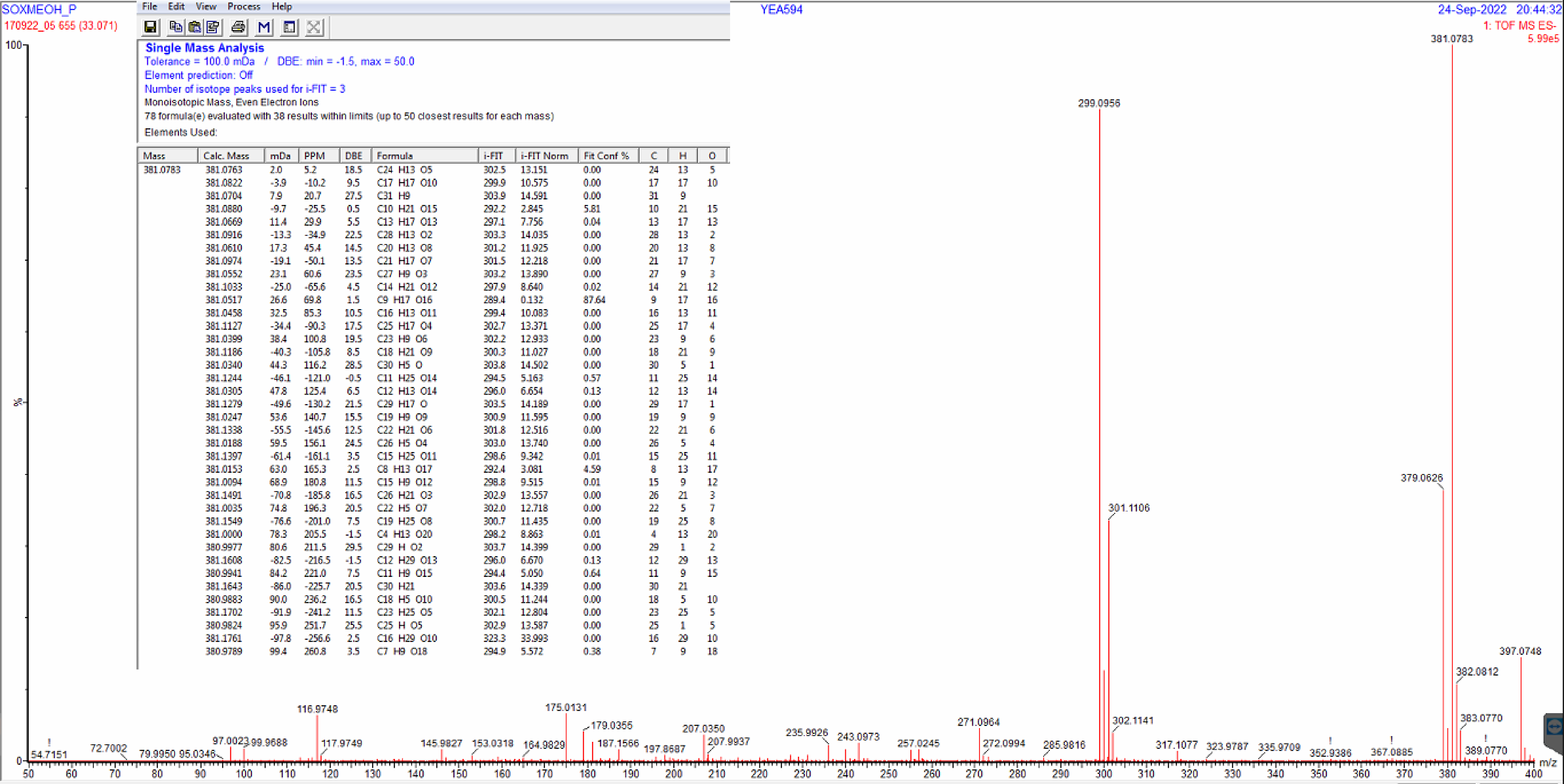

File Edit View Process Help

Single Mass Analysis

Tolerance = 100.0 mDa / DBE: min = -1.5, max = 50.0

Element prediction: Off

Number of isotope peaks used for i-FIT = 3

Monoisotopic Mass, Even Electron Ions

78 formula(e) evaluated with 38 results within limits (up to 50 closest results for each mass)

Elements Used:

Mass	Calc. Mass	mDa	PPM	DBE	Formula	i-FIT	i-FIT Norm	Fit Conf %	C	H	O
381.0783	381.0763	2.0	5.2	18.5	C24 H13 O5	302.5	13.151	0.00	24	13	5
	381.0822	-3.9	-10.2	9.5	C17 H17 O10	299.9	10.575	0.00	17	17	10
	381.0704	7.9	20.7	27.5	C31 H9	303.9	14.591	0.00	31	9	
	381.0880	-9.7	-25.5	0.5	C10 H21 O15	292.2	2.845	5.81	10	21	15
	381.0669	11.4	29.9	5.5	C13 H17 O13	297.1	7.756	0.04	13	17	13
	381.0916	-13.3	-34.9	22.5	C28 H13 O2	303.3	14.035	0.00	28	13	2
	381.0610	17.3	45.4	14.5	C20 H13 O8	301.2	11.925	0.00	20	13	8
	381.0974	-19.1	-50.1	13.5	C21 H17 O7	301.5	12.218	0.00	21	17	7
	381.0552	23.1	60.6	23.5	C27 H9 O3	303.2	13.890	0.00	27	9	3
	381.1033	-25.0	-65.6	4.5	C14 H21 O12	297.9	8.640	0.02	14	21	12
	381.0517	26.6	69.8	1.5	C9 H17 O16	289.4	0.132	87.64	9	17	16
	381.0458	32.5	85.3	10.5	C16 H13 O11	299.4	10.083	0.00	16	13	11
	381.1127	-34.4	-90.3	17.5	C25 H17 O4	302.7	13.371	0.00	25	17	4
	381.0399	38.4	100.8	19.5	C23 H9 O6	302.2	12.933	0.00	23	9	6
	381.1186	-40.3	-105.8	8.5	C18 H21 O9	300.3	11.027	0.00	18	21	9
	381.0340	44.3	116.2	28.5	C30 H5 O	303.8	14.502	0.00	30	5	1
	381.1244	-46.1	-121.0	-0.5	C11 H25 O14	294.5	5.163	0.57	11	25	14
	381.0305	47.8	125.4	6.5	C12 H13 O14	296.0	6.654	0.13	12	13	14
	381.1279	-49.6	-130.2	21.5	C29 H17 O	303.5	14.189	0.00	29	17	1
	381.0247	53.6	140.7	15.5	C19 H9 O9	300.9	11.595	0.00	19	9	9
	381.1338	-55.5	-145.6	12.5	C22 H21 O6	301.8	12.516	0.00	22	21	6
	381.0188	59.5	156.1	24.5	C26 H5 O4	303.0	13.740	0.00	26	5	4
	381.1397	-61.4	-161.1	3.5	C15 H25 O11	298.6	9.342	0.01	15	25	11
	381.0153	63.0	165.3	2.5	C8 H13 O17	292.4	3.081	4.59	8	13	17
	381.0094	68.9	180.8	11.5	C15 H9 O12	298.8	9.515	0.01	15	9	12
	381.1491	-70.8	-185.8	16.5	C26 H21 O3	302.9	13.557	0.00	26	21	3
	381.0035	74.8	196.3	20.5	C22 H5 O7	302.0	12.718	0.00	22	5	7
	381.1549	-76.6	-201.0	7.5	C19 H25 O8	300.7	11.435	0.00	19	25	8
	381.0000	78.3	205.5	-1.5	C4 H13 O20	298.2	8.863	0.01	4	13	20
	380.9977	80.6	211.5	29.5	C29 H O2	303.7	14.399	0.00	29	1	2
	381.1608	-82.5	-216.5	-1.5	C12 H29 O13	296.0	6.670	0.13	12	29	13
	380.9941	84.2	221.0	7.5	C11 H9 O15	294.4	5.050	0.64	11	9	15
	381.1643	-86.0	-225.7	20.5	C30 H21	303.6	14.339	0.00	30	21	
	380.9883	90.0	236.2	16.5	C18 H5 O10	300.5	11.244	0.00	18	5	10
	381.1702	-91.9	-241.2	11.5	C23 H25 O5	302.1	12.804	0.00	23	25	5
	380.9824	95.9	251.7	25.5	C25 H O5	302.9	13.587	0.00	25	1	5
	381.1761	-97.8	-256.6	2.5	C16 H29 O10	323.3	33.993	0.00	16	29	10
	380.9789	99.4	260.8	3.5	C7 H9 O18	294.9	5.572	0.38	7	9	18

Desktop 14:14

Spectrum - [170922_05]

File Edit Display Process Tools Window Help

SOXMEOH_P YEA594 24-Sep-2022 20:44:32

170922_05 663 (33.475) 1: TOF MS ES- 1.13e6

Single Mass Analysis

Tolerance = 100.0 mDa / DBE: min = -1.5, max = 50.0

Element prediction: Off

Number of isotope peaks used for i-FIT = 3

Monoisotopic Mass, Even Electron Ions

78 formula(e) evaluated with 38 results within limits (up to 50 closest results for each mass)

Elements Used:

Mass	Calc. Mass	mDa	PPM	DBE	Formula	i-FIT	i-FIT Norm	Fit Conf %	C	H	O
381.0775	381.0763	1.2	3.1	18.5	C24 H13 O5	342.3	11.482	0.00	24	13	5
	381.0822	-4.7	-12.3	9.5	C17 H17 O10	339.2	8.372	0.02	17	17	10
	381.0704	7.1	18.6	27.5	C31 H9	343.9	13.143	0.00	31	9	
	381.0880	-10.5	-27.6	0.5	C10 H21 O15	332.7	1.892	15.07	10	21	15
	381.0669	10.6	27.8	5.5	C13 H17 O13	335.3	4.505	1.11	13	17	13
	381.0916	-14.1	-37.0	22.5	C28 H13 O2	343.3	12.507	0.00	28	13	2
	381.0610	16.5	43.3	14.5	C20 H13 O8	340.8	10.029	0.00	20	13	8
	381.0974	-19.9	-52.2	13.5	C21 H17 O7	341.2	10.387	0.00	21	17	7
	381.0552	22.3	58.5	23.5	C27 H9 O3	343.1	12.338	0.00	27	9	3
	381.0517	25.8	67.7	1.5	C9 H17 O16	334.5	3.709	2.45	9	17	16
	381.1033	-25.8	-67.7	4.5	C14 H21 O12	336.6	5.823	0.30	14	21	12
	381.0458	31.7	83.2	10.5	C16 H13 O11	338.5	7.724	0.04	16	13	11
	381.1127	-35.2	-92.4	17.5	C25 H17 O4	342.5	11.741	0.00	25	17	4
	381.0399	37.6	98.7	19.5	C23 H9 O6	342.0	11.224	0.00	23	9	6
	381.1186	-41.1	-107.9	8.5	C18 H21 O9	339.8	8.948	0.01	18	21	9
	381.0340	43.5	114.2	28.5	C30 H5 O	343.8	13.039	0.00	30	5	1
	381.1244	-46.9	-123.1	-0.5	C11 H25 O14	332.4	1.565	20.91	11	25	14
	381.0305	47.0	123.3	6.5	C12 H13 O14	333.5	2.718	6.60	12	13	14
	381.1279	-50.4	-132.3	21.5	C29 H17 O	343.5	12.686	0.00	29	17	1
	381.0247	52.8	138.6	15.5	C19 H9 O9	340.4	9.622	0.01	19	9	9
	381.1338	-56.3	-147.7	12.5	C22 H21 O6	341.5	10.744	0.00	22	21	6
	381.0188	58.7	154.0	24.5	C26 H5 O4	343.0	12.161	0.00	26	5	4
	381.1397	-62.2	-163.2	3.5	C15 H25 O11	337.6	6.810	0.11	15	25	11
	381.0153	62.2	163.2	2.5	C8 H13 O17	336.1	5.329	0.48	8	13	17
	381.0094	68.1	178.7	11.5	C15 H9 O12	337.7	6.938	0.10	15	9	12
	381.1491	-71.6	-187.9	16.5	C26 H21 O3	342.8	11.961	0.00	26	21	3
	381.0035	74.0	194.2	20.5	C22 H5 O7	341.8	10.965	0.00	22	5	7
	381.1549	-77.4	-203.1	7.5	C19 H25 O8	340.3	9.458	0.01	19	25	8
	381.0000	77.5	203.4	-1.5	C4 H13 O20	339.5	8.681	0.02	4	13	20
	380.9977	79.8	209.4	29.5	C29 H O2	343.7	12.919	0.00	29	1	2
	381.1608	-83.3	-218.6	-1.5	C12 H29 O13	334.0	3.182	4.15	12	29	13
	380.9941	83.4	218.9	7.5	C11 H9 O15	331.5	0.725	48.45	11	9	15
	381.1643	-86.8	-227.8	20.5	C30 H21	343.7	12.860	0.00	30	21	
	380.9883	89.2	234.1	16.5	C18 H5 O10	340.0	9.176	0.01	18	5	10
	381.1702	-92.7	-243.3	11.5	C23 H25 O5	341.9	11.087	0.00	23	25	5
	380.9824	95.1	249.6	25.5	C25 H O5	342.8	11.982	0.00	25	1	5
	381.1761	-98.6	-258.7	2.5	C16 H29 O10	363.3	32.465	0.00	16	29	10
	380.9789	98.6	258.7	3.5	C7 H9 O18	337.4	6.620	0.13	7	9	18

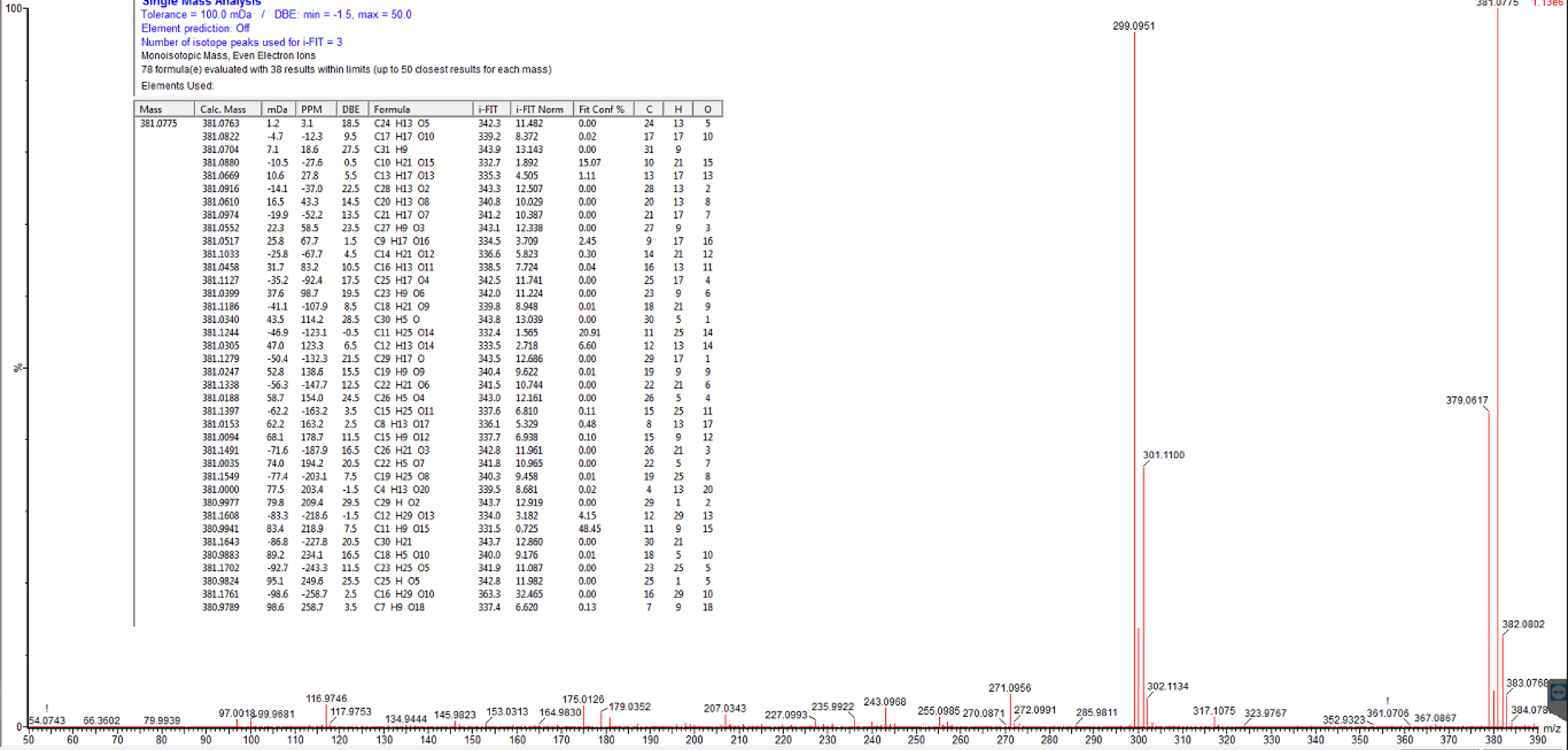

Desktop 14:17

Spectrum - [170922_05]

File Edit Display Process Tools Window Help

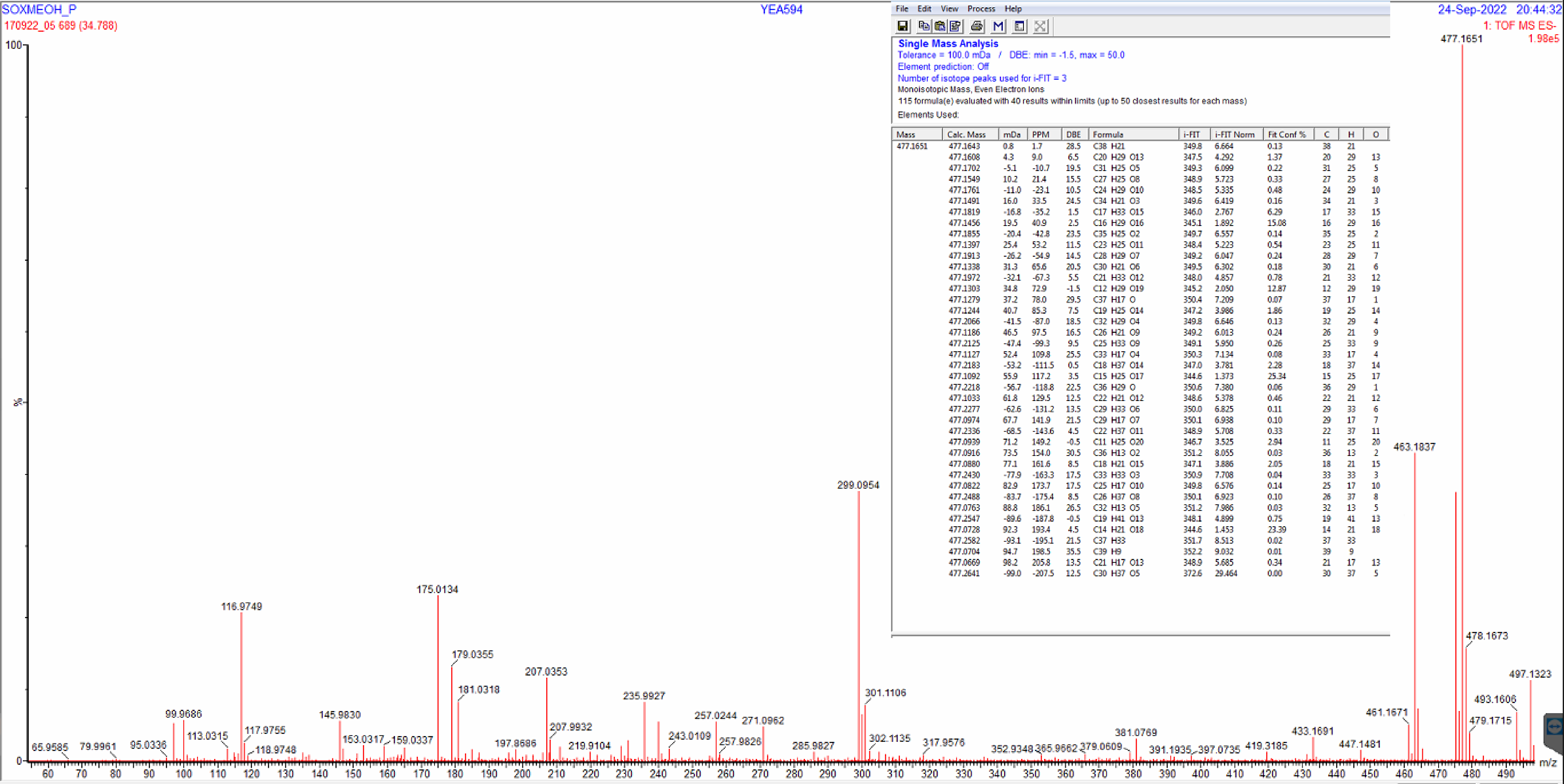

File Edit View Process Help

Single Mass Analysis

Tolerance = 100.0 mDa / DBE: min = -1.5, max = 50.0

Element prediction: Off

Number of isotope peaks used for i-FIT = 3

Monoisotopic Mass, Even Electron Ions

115 formula(e) evaluated with 40 results within limits (up to 50 closest results for each mass)

Elements Used:

Mass	Calc. Mass	mDa	PPM	DBE	Formula	i-FIT	i-FIT Norm	Fit Conf %	C	H	O
477.1651	477.1643	0.8	1.7	28.5	C38 H21	349.8	6.664	0.13	38	21	
	477.1608	4.3	9.0	6.5	C20 H29 O13	347.5	4.292	1.37	20	29	13
	477.1702	-5.1	-10.7	19.5	C31 H25 O5	349.3	6.099	0.22	31	25	5
	477.1549	10.2	21.4	15.5	C27 H25 O8	348.9	5.723	0.33	27	25	8
	477.1761	-11.0	-23.1	10.5	C24 H29 O10	348.5	5.335	0.48	24	29	10
	477.1491	16.0	33.5	24.5	C34 H21 O3	349.6	6.419	0.16	34	21	3
	477.1819	-16.8	-35.2	1.5	C17 H33 O15	346.0	2.767	6.29	17	33	15
	477.1456	19.5	40.9	2.5	C16 H29 O16	345.1	1.892	15.08	16	29	16
	477.1855	-20.4	-42.8	23.5	C35 H25 O2	349.7	6.557	0.14	35	25	2
	477.1397	25.4	53.2	11.5	C23 H25 O11	348.4	5.223	0.54	23	25	11
	477.1913	-26.2	-54.9	14.5	C28 H29 O7	349.2	6.047	0.24	28	29	7
	477.1338	31.3	65.6	20.5	C30 H21 O6	349.5	6.302	0.18	30	21	6
	477.1972	-32.1	-67.3	5.5	C21 H33 O12	348.0	4.857	0.78	21	33	12
	477.1303	34.8	72.9	-1.5	C12 H29 O19	345.2	2.050	12.87	12	29	19
	477.1279	37.2	78.0	29.5	C37 H17 O	350.4	7.209	0.07	37	17	1
	477.1244	40.7	85.3	7.5	C19 H25 O14	347.2	3.986	1.86	19	25	14
	477.2066	-41.5	-87.0	18.5	C32 H29 O4	349.8	6.646	0.13	32	29	4
	477.1186	46.5	97.5	16.5	C26 H21 O9	349.2	6.013	0.24	26	21	9
	477.2125	-47.4	-99.3	9.5	C25 H33 O9	349.1	5.950	0.26	25	33	9
	477.1127	52.4	109.8	25.5	C33 H17 O4	350.3	7.134	0.08	33	17	4
	477.2183	-53.2	-111.5	0.5	C18 H37 O14	347.0	3.781	2.28	18	37	14
	477.1092	55.9	117.2	3.5	C15 H25 O17	344.6	1.373	25.34	15	25	17
	477.2218	-56.7	-118.8	22.5	C36 H29 O	350.6	7.380	0.06	36	29	1
	477.1033	61.8	129.5	12.5	C22 H21 O12	348.6	5.378	0.46	22	21	12
	477.2277	-62.6	-131.2	13.5	C29 H33 O6	350.0	6.825	0.11	29	33	6
	477.0974	67.7	141.9	21.5	C29 H17 O7	350.1	6.938	0.10	29	17	7
	477.2336	-68.5	-143.6	4.5	C22 H37 O11	348.9	5.708	0.33	22	37	11
	477.0939	71.2	149.2	-0.5	C11 H25 O20	346.7	3.525	2.94	11	25	20
	477.0916	73.5	154.0	30.5	C36 H13 O2	351.2	8.055	0.03	36	13	2
	477.0880	77.1	161.6	8.5	C18 H21 O15	347.1	3.886	2.05	18	21	15
	477.2430	-77.9	-163.3	17.5	C33 H33 O3	350.9	7.708	0.04	33	33	3
	477.0822	82.9	173.7	17.5	C25 H17 O10	349.8	6.576	0.14	25	17	10
	477.2488	-83.7	-175.4	8.5	C26 H37 O8	350.1	6.923	0.10	26	37	8
	477.0763	88.8	186.1	26.5	C32 H13 O5	351.2	7.986	0.03	32	13	5
	477.2547	-89.6	-187.8	-0.5	C19 H41 O13	348.1	4.899	0.75	19	41	13
	477.0728	92.3	193.4	4.5	C14 H21 O18	344.6	1.453	23.39	14	21	18
	477.2582	-93.1	-195.1	21.5	C37 H33	351.7	8.513	0.02	37	33	
	477.0704	94.7	198.5	35.5	C39 H9	352.2	9.032	0.01	39	9	
	477.0669	98.2	205.8	13.5	C21 H17 O13	348.9	5.685	0.34	21	17	13
	477.2641	-99.0	-207.5	12.5	C30 H37 O5	372.6	29.464	0.00	30	37	5

Desktop 14:19

Spectrum - [170922_05]

File Edit Display Process Tools Window Help

SOXMEOH_P YEA594 24-Sep-2022 20:44:32

170922_05 741 (37.414) 1: TOF MS ES-

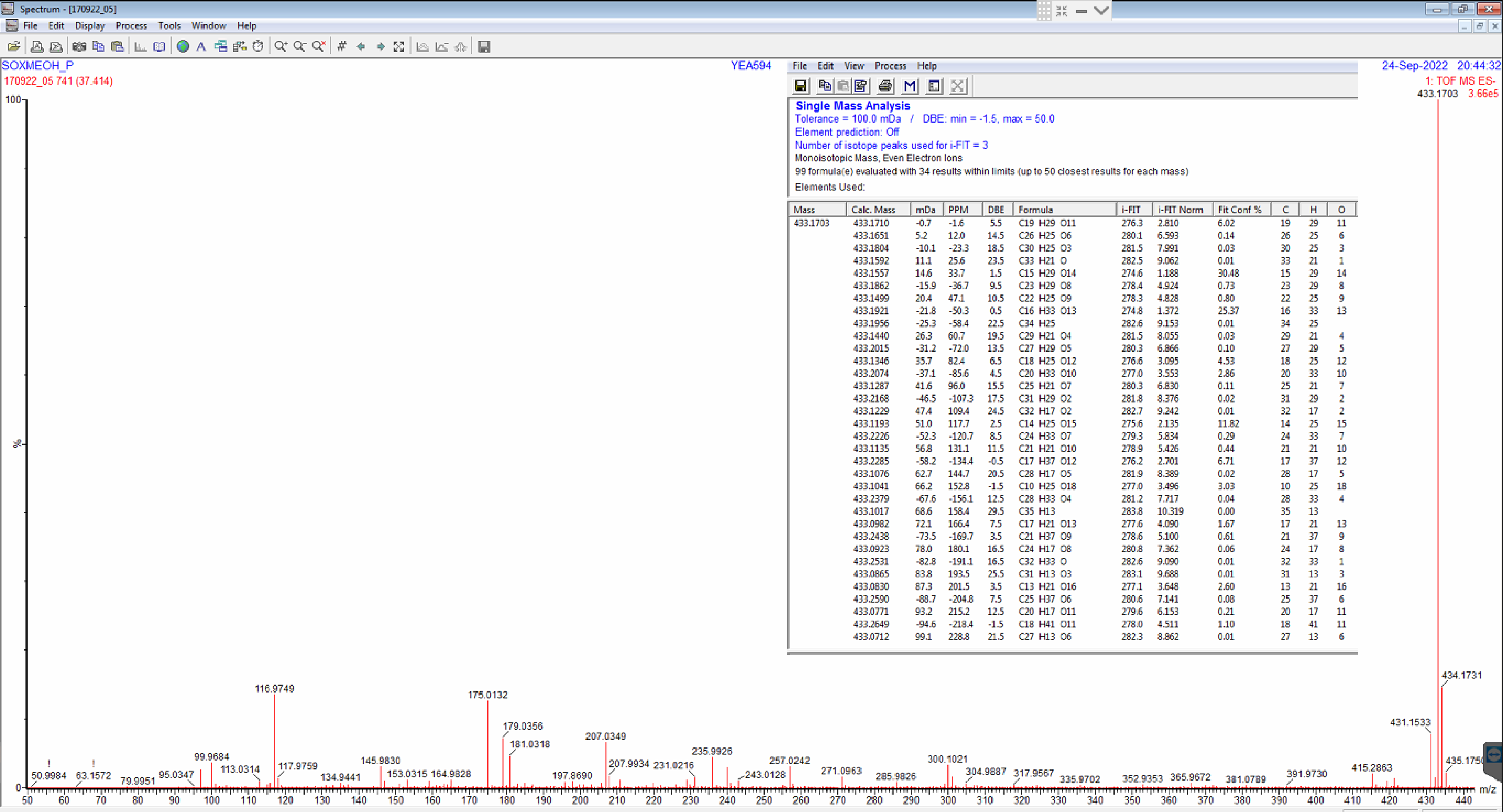

File Edit View Process Help

Single Mass Analysis

Tolerance = 100.0 mDa / DBE: min = -1.5, max = 50.0

Element prediction: Off

Number of isotope peaks used for i-FIT = 3

Monoisotopic Mass, Even Electron Ions

99 formula(e) evaluated with 34 results within limits (up to 50 closest results for each mass)

Elements Used:

Mass	Calc. Mass	mDa	PPM	DBE	Formula	i-FIT	i-FIT Norm	Fit Conf %	C	H	O
433.1703	433.1710	-0.7	-1.6	5.5	C19 H29 O11	276.3	2.810	6.02	19	29	11
	433.1651	5.2	12.0	14.5	C26 H25 O6	280.1	6.593	0.14	26	25	6
	433.1804	-10.1	-23.3	18.5	C30 H25 O3	281.5	7.991	0.03	30	25	3
	433.1592	11.1	25.6	23.5	C33 H21 O	282.5	9.062	0.01	33	21	1
	433.1557	14.6	33.7	1.5	C15 H29 O14	274.6	1.188	30.48	15	29	14
	433.1862	-15.9	-36.7	9.5	C23 H29 O8	278.4	4.924	0.73	23	29	8
	433.1499	20.4	47.1	10.5	C22 H25 O9	278.3	4.828	0.80	22	25	9
	433.1921	-21.8	-50.3	0.5	C16 H33 O13	274.8	1.372	25.37	16	33	13
	433.1956	-25.3	-58.4	22.5	C34 H25	282.6	9.153	0.01	34	25	
	433.1440	26.3	60.7	19.5	C29 H21 O4	281.5	8.055	0.03	29	21	4
	433.2015	-31.2	-72.0	13.5	C27 H29 O5	280.3	6.866	0.10	27	29	5
	433.1346	35.7	82.4	6.5	C18 H25 O12	276.6	3.095	4.53	18	25	12
	433.2074	-37.1	-85.6	4.5	C20 H33 O10	277.0	3.553	2.86	20	33	10
	433.1287	41.6	96.0	15.5	C25 H21 O7	280.3	6.830	0.11	25	21	7
	433.2168	-46.5	-107.3	17.5	C31 H29 O2	281.8	8.376	0.02	31	29	2
	433.1229	47.4	109.4	24.5	C32 H17 O2	282.7	9.242	0.01	32	17	2
	433.1193	51.0	117.7	2.5	C14 H25 O15	275.6	2.135	11.82	14	25	15
	433.2226	-52.3	-120.7	8.5	C24 H33 O7	279.3	5.834	0.29	24	33	7
	433.1135	56.8	131.1	11.5	C21 H21 O10	278.9	5.426	0.44	21	21	10
	433.2285	-58.2	-134.4	-0.5	C17 H37 O12	276.2	2.701	6.71	17	37	12
	433.1076	62.7	144.7	20.5	C28 H17 O5	281.9	8.389	0.02	28	17	5
	433.1041	66.2	152.8	-1.5	C10 H25 O18	277.0	3.496	3.03	10	25	18
	433.2379	-67.6	-156.1	12.5	C28 H33 O4	281.2	7.717	0.04	28	33	4
	433.1017	68.6	158.4	29.5	C35 H13	283.8	10.319	0.00	35	13	
	433.0982	72.1	166.4	7.5	C17 H21 O13	277.6	4.090	1.67	17	21	13
	433.2438	-73.5	-169.7	3.5	C21 H37 O9	278.6	5.100	0.61	21	37	9
	433.0923	78.0	180.1	16.5	C24 H17 O8	280.8	7.362	0.06	24	17	8
	433.2531	-82.8	-191.1	16.5	C32 H33 O	282.6	9.090	0.01	32	33	1
	433.0865	83.8	193.5	25.5	C31 H13 O3	283.1	9.688	0.01	31	13	3
	433.0830	87.3	201.5	3.5	C13 H21 O16	277.1	3.648	2.60	13	21	16
	433.2590	-88.7	-204.8	7.5	C25 H37 O6	280.6	7.141	0.08	25	37	6
	433.0771	93.2	215.2	12.5	C20 H17 O11	279.6	6.153	0.21	20	17	11
	433.2649	-94.6	-218.4	-1.5	C18 H41 O11	278.0	4.511	1.10	18	41	11
	433.0712	99.1	228.8	21.5	C27 H13 O6	282.3	8.862	0.01	27	13	6

Desktop 14:22

Spectrum - [170922_05]

File Edit Display Process Tools Window Help

SOXMEOH_P YEA594 24-Sep-2022 20:44:32

170922_05 797 (40.242) 1: TOF MS ES- 1.18e6

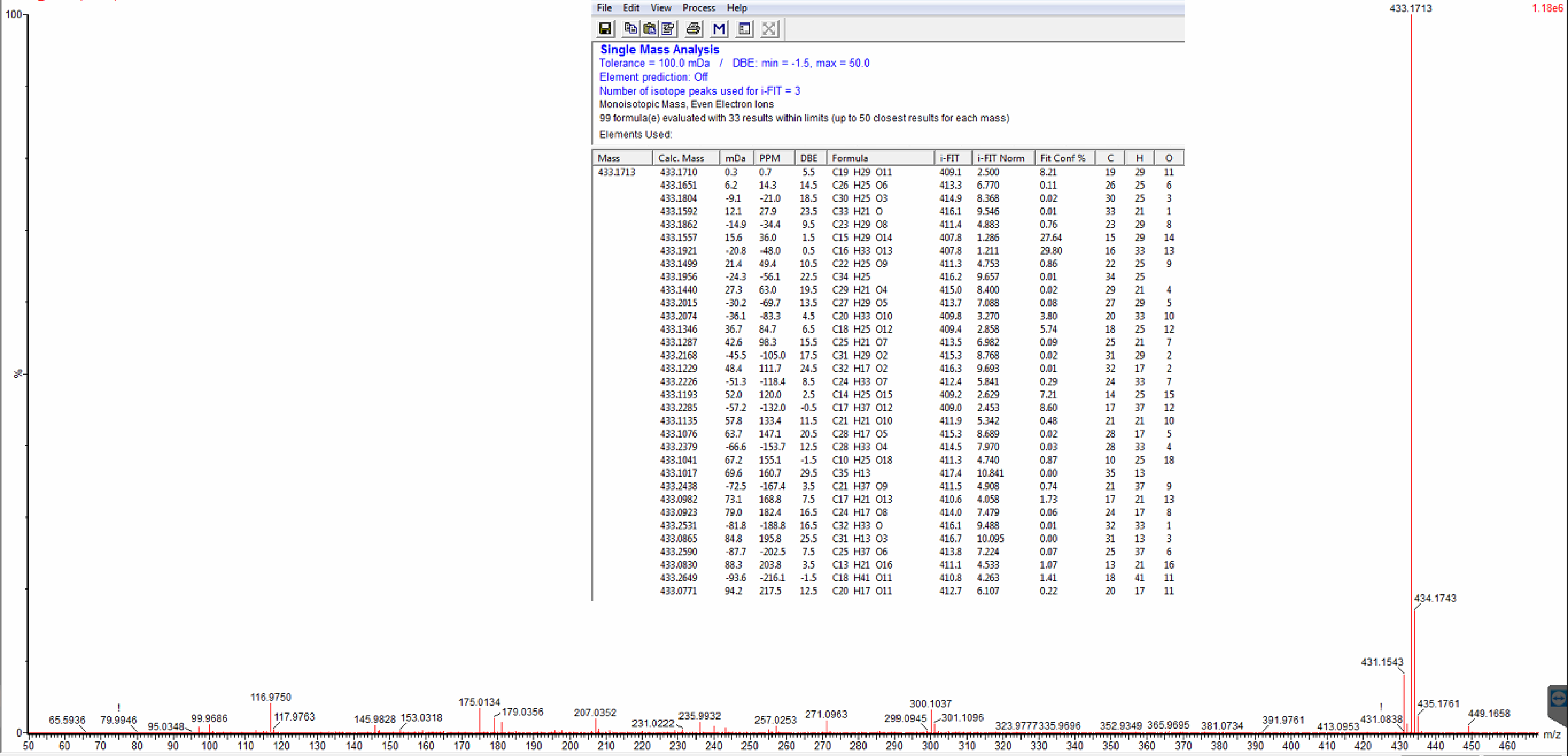

File Edit View Process Help

Single Mass Analysis

Tolerance = 100.0 mDa / DBE: min = -1.5, max = 50.0

Element prediction: Off

Number of isotope peaks used for i-FIT = 3

Monoisotopic Mass, Even Electron Ions

99 formula(e) evaluated with 33 results within limits (up to 50 closest results for each mass)

Elements Used:

Mass	Calc. Mass	mDa	PPM	DBE	Formula	i-FIT	i-FIT Norm	Fit Conf %	C	H	O
433.1713	433.1710	0.3	0.7	5.5	C19 H29 O11	409.1	2.500	8.21	19	29	11
	433.1651	6.2	14.3	14.5	C26 H25 O6	413.3	6.770	0.11	26	25	6
	433.1804	-9.1	-21.0	18.5	C30 H25 O3	414.9	8.368	0.02	30	25	3
	433.1592	12.1	27.9	23.5	C33 H21 O	416.1	9.546	0.01	33	21	1
	433.1862	-14.9	-34.4	9.5	C23 H29 O8	411.4	4.883	0.76	23	29	8
	433.1557	15.6	36.0	1.5	C15 H29 O14	407.8	1.286	27.64	15	29	14
	433.1921	-20.8	-48.0	0.5	C16 H33 O13	407.8	1.211	29.80	16	33	13
	433.1499	21.4	49.4	10.5	C22 H25 O9	411.3	4.753	0.86	22	25	9
	433.1956	-24.3	-56.1	22.5	C34 H25	416.2	9.657	0.01	34	25	
	433.1440	27.3	63.0	19.5	C29 H21 O4	415.0	8.400	0.02	29	21	4
	433.2015	-30.2	-69.7	13.5	C27 H29 O5	413.7	7.088	0.08	27	29	5
	433.2074	-36.1	-83.3	4.5	C20 H33 O10	409.8	3.270	3.80	20	33	10
	433.1346	36.7	84.7	6.5	C18 H25 O12	409.4	2.858	5.74	18	25	12
	433.1287	42.6	98.3	15.5	C25 H21 O7	413.5	6.982	0.09	25	21	7
	433.2168	-45.5	-105.0	17.5	C31 H29 O2	415.3	8.768	0.02	31	29	2
	433.1229	48.4	111.7	24.5	C32 H17 O2	416.3	9.693	0.01	32	17	2
	433.2226	-51.3	-118.4	8.5	C24 H33 O7	412.4	5.841	0.29	24	33	7
	433.1193	52.0	120.0	2.5	C14 H25 O15	409.2	2.629	7.21	14	25	15
	433.2285	-57.2	-132.0	-0.5	C17 H37 O12	409.0	2.453	8.60	17	37	12
	433.1135	57.8	133.4	11.5	C21 H21 O10	411.9	5.342	0.48	21	21	10
	433.1076	63.7	147.1	20.5	C28 H17 O5	415.3	8.689	0.02	28	17	5
	433.2379	-66.6	-153.7	12.5	C28 H33 O4	414.5	7.970	0.03	28	33	4
	433.1041	67.2	155.1	-1.5	C10 H25 O18	411.3	4.740	0.87	10	25	18
	433.1017	69.6	160.7	29.5	C35 H13	417.4	10.841	0.00	35	13	
	433.2438	-72.5	-167.4	3.5	C21 H37 O9	411.5	4.908	0.74	21	37	9
	433.0982	73.1	168.8	7.5	C17 H21 O13	410.6	4.058	1.73	17	21	13
	433.0923	79.0	182.4	16.5	C24 H17 O8	414.0	7.479	0.06	24	17	8
	433.2531	-81.8	-188.8	16.5	C32 H33 O	416.1	9.488	0.01	32	33	1
	433.0865	84.8	195.8	25.5	C31 H13 O3	416.7	10.095	0.00	31	13	3
	433.2590	-87.7	-202.5	7.5	C25 H37 O6	413.8	7.224	0.07	25	37	6
	433.0830	88.3	203.8	3.5	C13 H21 O16	411.1	4.533	1.07	13	21	16
	433.2649	-93.6	-216.1	-1.5	C18 H41 O11	410.8	4.263	1.41	18	41	11
	433.0771	94.2	217.5	12.5	C20 H17 O11	412.7	6.107	0.22	20	17	11

Desktop 14:25

Spectrum - [170922_05]

SOXMEOH_P

170922_05 808 (40.798)

YEA594

24-Sep-2022 20:44:32

1: TOF MS ES-
8.50e5

Single Mass Analysis

Tolerance = 100.0 mDa / DBE: min = -1.5, max = 50.0

Element prediction: Off

Number of isotope peaks used for i-FIT = 3

Monoisotopic Mass, Even Electron Ions

99 formula(e) evaluated with 34 results within limits (up to 50 closest results for each mass)

Elements Used:

Mass	Calc. Mass	mDa	PPM	DBE	Formula	i-FIT	i-FIT Norm	Fit Conf %	C	H	O
433.1707	433.1710	-0.3	-0.7	5.5	C19 H29 O11	347.8	2.966	5.15	19	29	11
	433.1651	5.6	12.9	14.5	C26 H25 O6	352.0	7.154	0.08	26	25	6
	433.1804	-9.7	-22.4	18.5	C30 H25 O3	353.5	8.623	0.02	30	25	3
	433.1592	11.5	26.5	23.5	C33 H21 O	354.5	9.693	0.01	33	21	1
	433.1557	15.0	34.6	1.5	C15 H29 O14	346.0	1.180	30.74	15	29	14
	433.1862	-15.5	-35.8	9.5	C23 H29 O8	350.3	5.402	0.45	23	29	8
	433.1499	20.8	48.0	10.5	C22 H25 O9	350.1	5.233	0.53	22	25	9
	433.1921	-21.4	-49.4	0.5	C16 H33 O13	346.1	1.210	29.83	16	33	13
	433.1956	-24.9	-57.5	22.5	C34 H25	354.7	9.803	0.01	34	25	
	433.1440	26.7	61.6	19.5	C29 H21 O4	353.5	8.615	0.02	29	21	4
	433.2015	-30.8	-71.1	13.5	C27 H29 O5	352.3	7.436	0.06	27	29	5
	433.1346	36.1	83.3	6.5	C18 H25 O12	348.0	3.162	4.24	18	25	12
	433.2074	-36.7	-84.7	4.5	C20 H33 O10	348.5	3.682	2.52	20	33	10
	433.1287	42.0	97.0	15.5	C25 H21 O7	352.1	7.258	0.07	25	21	7
	433.2168	-46.1	-106.4	17.5	C31 H29 O2	353.8	8.959	0.01	31	29	2
	433.1229	47.8	110.3	24.5	C32 H17 O2	354.6	9.785	0.01	32	17	2
	433.1193	51.4	118.7	2.5	C14 H25 O15	347.2	2.349	9.55	14	25	15
	433.2226	-51.9	-119.8	8.5	C24 H33 O7	351.0	6.191	0.20	24	33	7
	433.1135	57.2	132.0	11.5	C21 H21 O10	350.5	5.609	0.37	21	21	10
	433.2285	-57.8	-133.4	-0.5	C17 H37 O12	347.3	2.460	8.54	17	37	12
	433.1076	63.1	145.7	20.5	C28 H17 O5	353.7	8.807	0.01	28	17	5
	433.1041	66.6	153.8	-1.5	C10 H25 O18	348.9	4.069	1.71	10	25	18
	433.2379	-67.2	-155.1	12.5	C28 H33 O4	353.0	8.157	0.03	28	33	4
	433.1017	69.0	159.3	29.5	C35 H13	355.7	10.836	0.00	35	13	
	433.0982	72.5	167.4	7.5	C17 H21 O13	348.9	4.090	1.67	17	21	13
	433.2438	-73.1	-168.8	3.5	C21 H37 O9	350.0	5.115	0.60	21	37	9
	433.0923	78.4	181.0	16.5	C24 H17 O8	352.5	7.604	0.05	24	17	8
	433.2531	-82.4	-190.2	16.5	C32 H33 O	354.4	9.557	0.01	32	33	1
	433.0865	84.2	194.4	25.5	C31 H13 O3	355.0	10.102	0.00	31	13	3
	433.0830	87.7	202.5	3.5	C13 H21 O16	348.9	4.062	1.72	13	21	16
	433.2590	-88.3	-203.8	7.5	C25 H37 O6	352.2	7.352	0.06	25	37	6
	433.0771	93.6	216.1	12.5	C20 H17 O11	351.0	6.173	0.21	20	17	11
	433.2649	-94.2	-217.5	-1.5	C18 H41 O11	349.0	4.189	1.52	18	41	11
	433.0712	99.5	229.7	21.5	C27 H13 O6	354.0	9.142	0.01	27	13	6

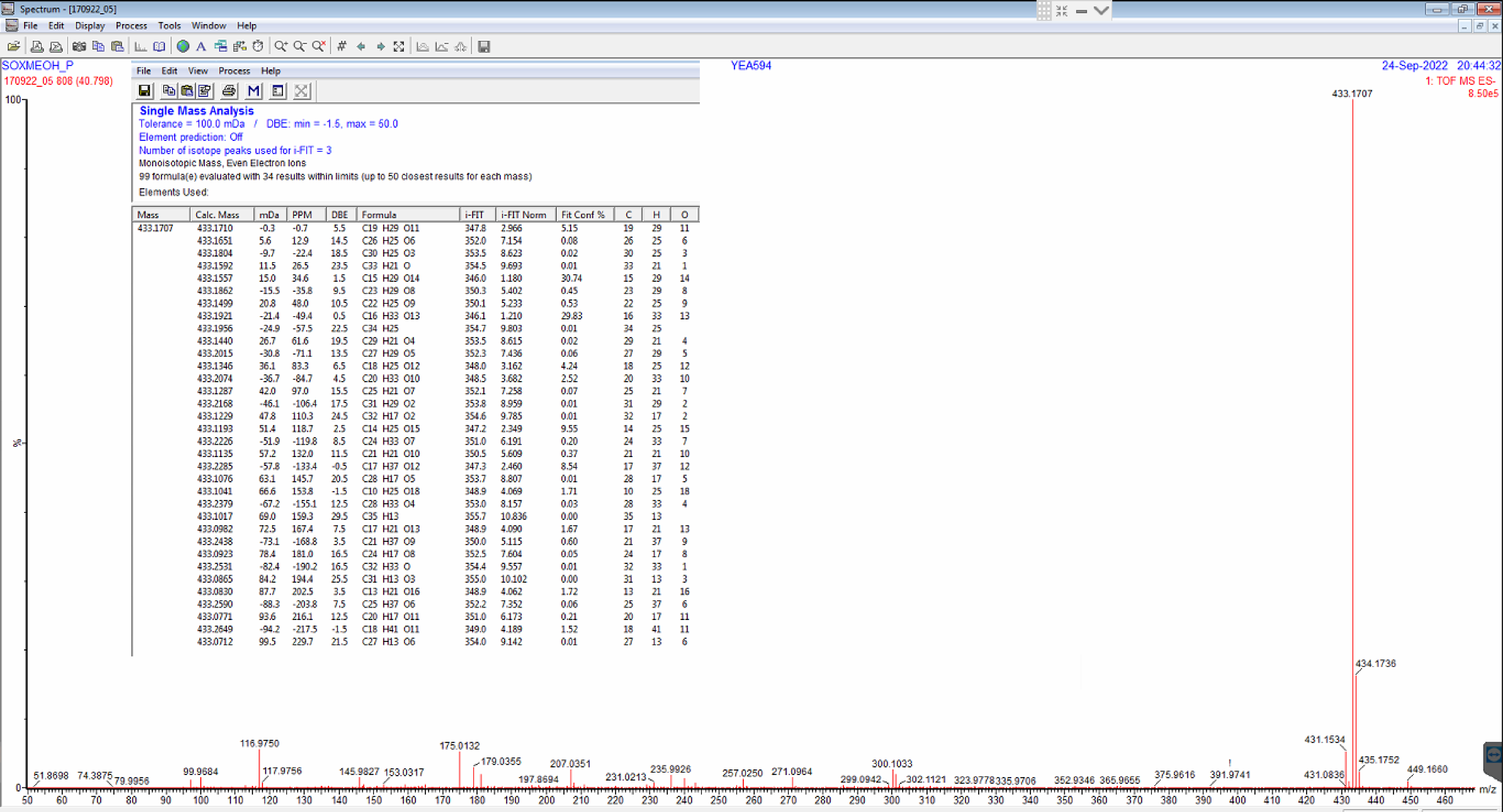

Spectrum - [170922_05]

File Edit Display Process Tools Window Help

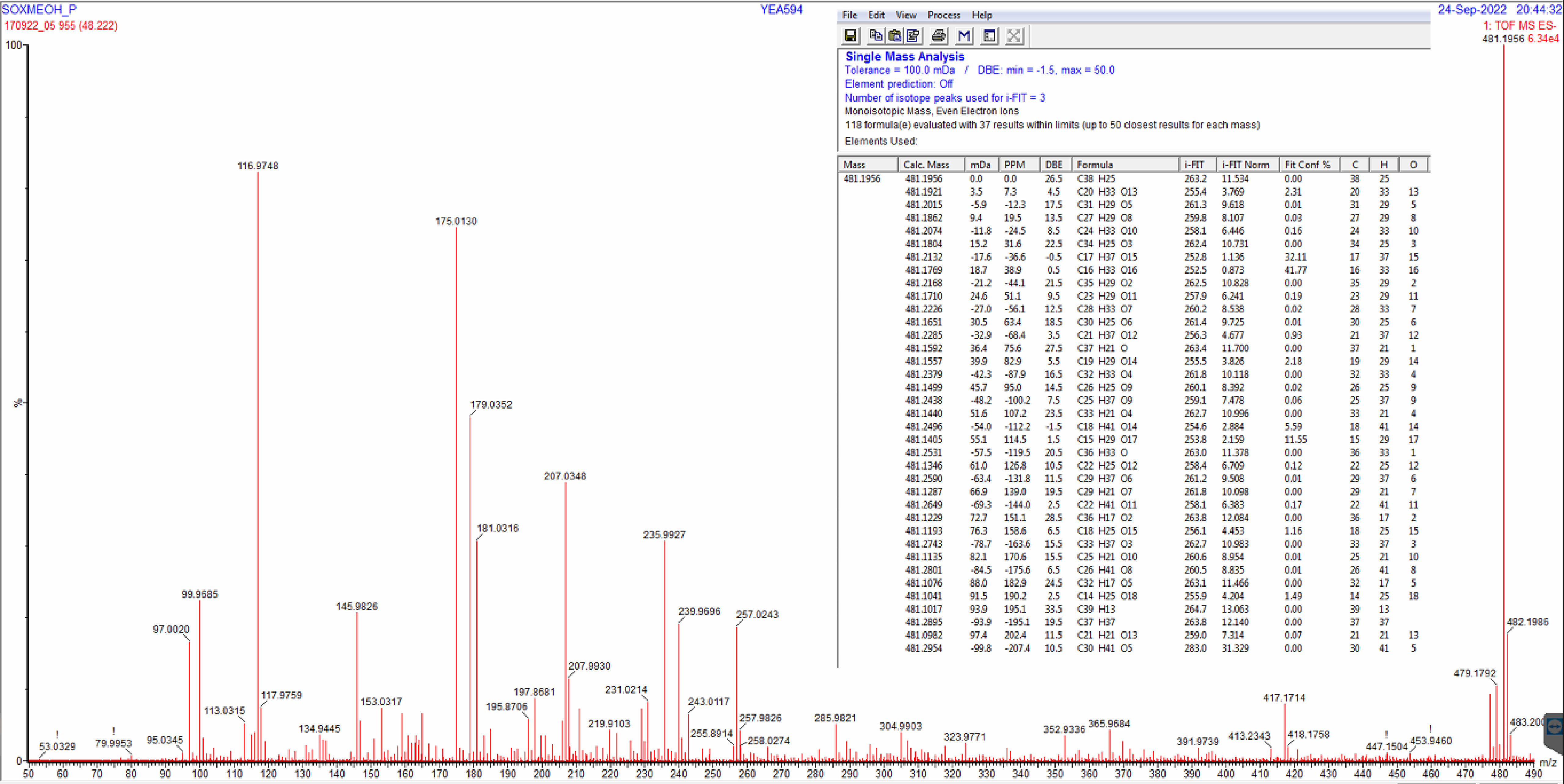

File Edit View Process Help

Single Mass Analysis

Tolerance = 100.0 mDa / DBE: min = -1.5, max = 50.0

Element prediction: Off

Number of isotope peaks used for i-FIT = 3

Monoisotopic Mass, Even Electron Ions

118 formula(e) evaluated with 37 results within limits (up to 50 closest results for each mass)

Elements Used:

Mass	Calc. Mass	mDa	PPM	DBE	Formula	i-FIT	i-FIT Norm	Fit Conf %	C	H	O
481.1956	481.1956	0.0	0.0	26.5	C38 H25	263.2	11.534	0.00	38	25	
	481.1921	3.5	7.3	4.5	C20 H33 O13	255.4	3.769	2.31	20	33	13
	481.2015	-5.9	-12.3	17.5	C31 H29 O5	261.3	9.618	0.01	31	29	5
	481.1862	9.4	19.5	13.5	C27 H29 O8	259.8	8.107	0.03	27	29	8
	481.2074	-11.8	-24.5	8.5	C24 H33 O10	258.1	6.446	0.16	24	33	10
	481.1804	15.2	31.6	22.5	C34 H25 O3	262.4	10.731	0.00	34	25	3
	481.2132	-17.6	-36.6	-0.5	C17 H37 O15	252.8	1.136	32.11	17	37	15
	481.1769	18.7	38.9	0.5	C16 H33 O16	252.5	0.873	41.77	16	33	16
	481.2168	-21.2	-44.1	21.5	C35 H29 O2	262.5	10.828	0.00	35	29	2
	481.1710	24.6	51.1	9.5	C23 H29 O11	257.9	6.241	0.19	23	29	11
	481.2226	-27.0	-56.1	12.5	C28 H33 O7	260.2	8.538	0.02	28	33	7
	481.1651	30.5	63.4	18.5	C30 H25 O6	261.4	9.725	0.01	30	25	6
	481.2285	-32.9	-68.4	3.5	C21 H37 O12	256.3	4.677	0.93	21	37	12
	481.1592	36.4	75.6	27.5	C37 H21 O	263.4	11.700	0.00	37	21	1
	481.1557	39.9	82.9	5.5	C19 H29 O14	255.5	3.826	2.18	19	29	14
	481.2379	-42.3	-87.9	16.5	C32 H33 O4	261.8	10.118	0.00	32	33	4
	481.1499	45.7	95.0	14.5	C26 H25 O9	260.1	8.392	0.02	26	25	9
	481.2438	-48.2	-100.2	7.5	C25 H37 O9	259.1	7.478	0.06	25	37	9
	481.1440	51.6	107.2	23.5	C33 H21 O4	262.7	10.996	0.00	33	21	4
	481.2496	-54.0	-112.2	-1.5	C18 H41 O14	254.6	2.884	5.59	18	41	14
	481.1405	55.1	114.5	1.5	C15 H29 O17	253.8	2.159	11.55	15	29	17
	481.2531	-57.5	-119.5	20.5	C36 H33 O	263.0	11.378	0.00	36	33	1
	481.1346	61.0	126.8	10.5	C22 H25 O12	258.4	6.709	0.12	22	25	12
	481.2590	-63.4	-131.8	11.5	C29 H37 O6	261.2	9.508	0.01	29	37	6
	481.1287	66.9	139.0	19.5	C29 H21 O7	261.8	10.098	0.00	29	21	7
	481.2649	-69.3	-144.0	2.5	C22 H41 O11	258.1	6.383	0.17	22	41	11
	481.1229	72.7	151.1	28.5	C36 H17 O2	263.8	12.084	0.00	36	17	2
	481.1193	76.3	158.6	6.5	C18 H25 O15	256.1	4.453	1.16	18	25	15
	481.2743	-78.7	-163.6	15.5	C33 H37 O3	262.7	10.983	0.00	33	37	3
	481.1135	82.1	170.6	15.5	C25 H21 O10	260.6	8.954	0.01	25	21	10
	481.2801	-84.5	-175.6	6.5	C26 H41 O8	260.5	8.835	0.01	26	41	8
	481.1076	88.0	182.9	24.5	C32 H17 O5	263.1	11.466	0.00	32	17	5
	481.1041	91.5	190.2	2.5	C14 H25 O18	255.9	4.204	1.49	14	25	18
	481.1017	93.9	195.1	33.5	C39 H13	264.7	13.063	0.00	39	13	
	481.2895	-93.9	-195.1	19.5	C37 H37	263.8	12.140	0.00	37	37	
	481.0982	97.4	202.4	11.5	C21 H21 O13	259.0	7.314	0.07	21	21	13
	481.2954	-99.8	-207.4	10.5	C30 H41 O5	283.0	31.329	0.00	30	41	5

Desktop 14:30

Spectrum - [170922_05]

File Edit Display Process Tools Window Help

SOXMEOH_P YEA594 24-Sep-2022 20:44:32

170922_05 1130 (57.060) 1: TOF MS ES- 5.63e5

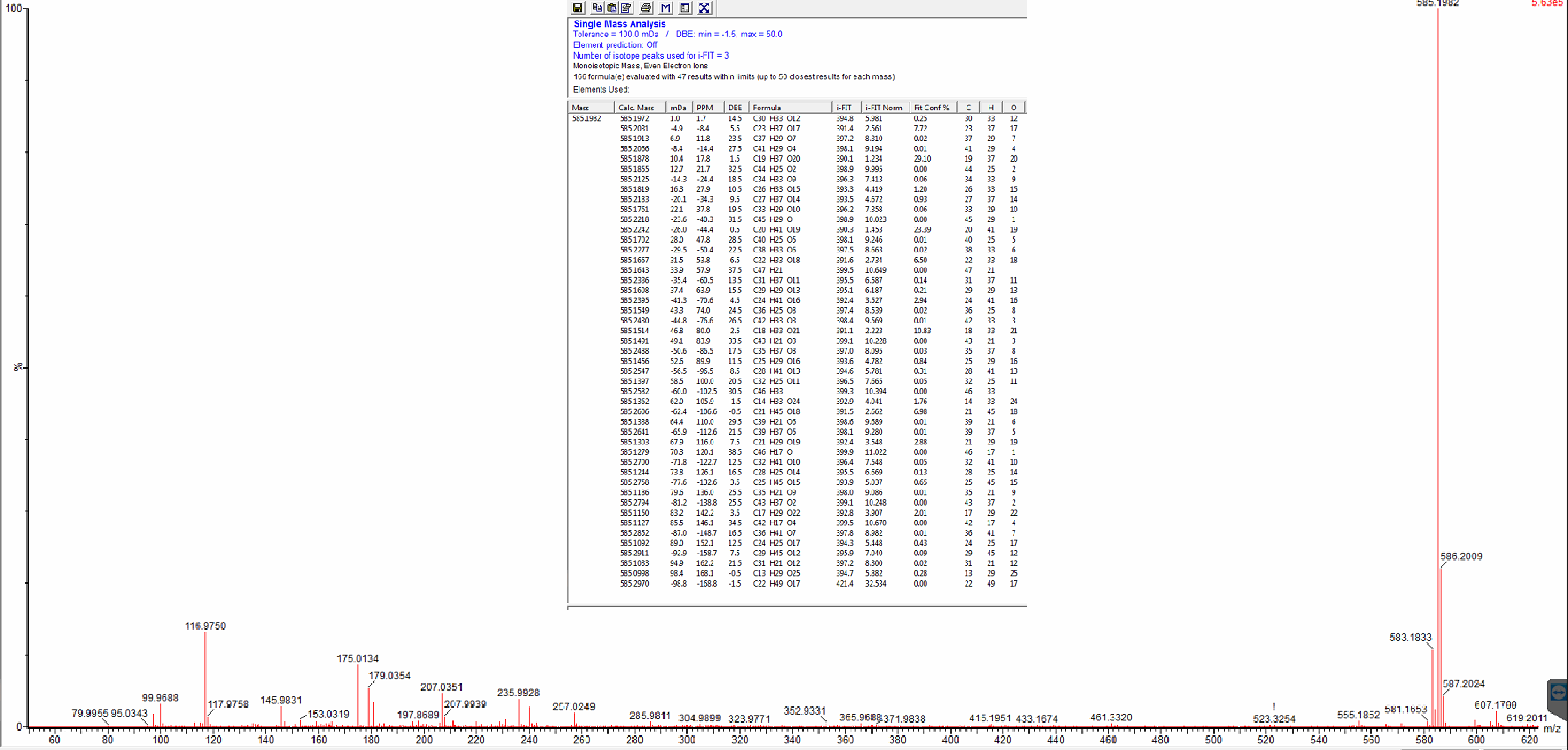

File Edit View Process Help

Single Mass Analysis

Tolerance = 100.0 mDa / DBE: min = -1.5, max = 50.0

Element prediction: Off

Number of isotope peaks used for i-FIT = 3

Monoisotopic Mass, Even Electron Ions

166 formula(e) evaluated with 47 results within limits (up to 50 closest results for each mass)

Elements Used:

Mass	Calc. Mass	mDa	PPM	DBE	Formula	i-FIT	i-FIT Norm	Fit Conf %	C	H	O
585.1982	585.1972	1.0	1.7	14.5	C30 H33 O12	394.8	5.981	0.25	30	33	12
	585.2031	-4.9	-8.4	5.5	C23 H37 O17	391.4	2.561	7.72	23	37	17
	585.1913	6.9	11.8	23.5	C37 H29 O7	397.2	8.310	0.02	37	29	7
	585.2066	-8.4	-14.4	27.5	C41 H29 O4	398.1	9.194	0.01	41	29	4
	585.1878	10.4	17.8	1.5	C19 H37 O20	390.1	1.234	29.10	19	37	20
	585.1855	12.7	21.7	32.5	C44 H25 O2	398.9	9.995	0.00	44	25	2
	585.2125	-14.3	-24.4	18.5	C34 H33 O9	396.3	7.413	0.06	34	33	9
	585.1819	16.3	27.9	10.5	C26 H33 O15	393.3	4.419	1.20	26	33	15
	585.2183	-20.1	-34.3	9.5	C27 H37 O14	393.5	4.672	0.93	27	37	14
	585.1761	22.1	37.8	19.5	C33 H29 O10	396.2	7.358	0.06	33	29	10
	585.2218	-23.6	-40.3	31.5	C45 H29 O	398.9	10.023	0.00	45	29	1
	585.2242	-26.0	-44.4	0.5	C20 H41 O19	390.3	1.453	23.39	20	41	19
	585.1702	28.0	47.8	28.5	C40 H25 O5	398.1	9.246	0.01	40	25	5
	585.2277	-29.5	-50.4	22.5	C38 H33 O6	397.5	8.663	0.02	38	33	6
	585.1667	31.5	53.8	6.5	C22 H33 O18	391.6	2.734	6.50	22	33	18
	585.1643	33.9	57.9	37.5	C47 H21	399.5	10.649	0.00	47	21	
	585.2336	-35.4	-60.5	13.5	C31 H37 O11	395.5	6.587	0.14	31	37	11
	585.1608	37.4	63.9	15.5	C29 H29 O13	395.1	6.187	0.21	29	29	13
	585.2395	-41.3	-70.6	4.5	C24 H41 O16	392.4	3.527	2.94	24	41	16
	585.1549	43.3	74.0	24.5	C36 H25 O8	397.4	8.539	0.02	36	25	8
	585.2430	-44.8	-76.6	26.5	C42 H33 O3	398.4	9.569	0.01	42	33	3
	585.1514	46.8	80.0	2.5	C18 H33 O21	391.1	2.223	10.83	18	33	21
	585.1491	49.1	83.9	33.5	C43 H21 O3	399.1	10.228	0.00	43	21	3
	585.2488	-50.6	-86.5	17.5	C35 H37 O8	397.0	8.095	0.03	35	37	8
	585.1456	52.6	89.9	11.5	C25 H29 O16	393.6	4.782	0.84	25	29	16
	585.2547	-56.5	-96.5	8.5	C28 H41 O13	394.6	5.781	0.31	28	41	13
	585.1397	58.5	100.0	20.5	C32 H25 O11	396.5	7.665	0.05	32	25	11
	585.2582	-60.0	-102.5	30.5	C46 H33	399.3	10.394	0.00	46	33	
	585.1362	62.0	105.9	-1.5	C14 H33 O24	392.9	4.041	1.76	14	33	24
	585.2606	-62.4	-106.6	-0.5	C21 H45 O18	391.5	2.662	6.98	21	45	18
	585.1338	64.4	110.0	29.5	C39 H21 O6	398.6	9.689	0.01	39	21	6
	585.2641	-65.9	-112.6	21.5	C39 H37 O5	398.1	9.280	0.01	39	37	5
	585.1303	67.9	116.0	7.5	C21 H29 O19	392.4	3.548	2.88	21	29	19
	585.1279	70.3	120.1	38.5	C46 H17 O	399.9	11.022	0.00	46	17	1
	585.2700	-71.8	-122.7	12.5	C32 H41 O10	396.4	7.548	0.05	32	41	10
	585.1244	73.8	126.1	16.5	C28 H25 O14	395.5	6.669	0.13	28	25	14
	585.2758	-77.6	-132.6	3.5	C25 H45 O15	393.9	5.037	0.65	25	45	15
	585.1186	79.6	136.0	25.5	C35 H21 O9	398.0	9.086	0.01	35	21	9
	585.2794	-81.2	-138.8	25.5	C43 H37 O2	399.1	10.248	0.00	43	37	2
	585.1150	83.2	142.2	3.5	C17 H29 O22	392.8	3.907	2.01	17	29	22
	585.1127	85.5	146.1	34.5	C42 H17 O4	399.5	10.670	0.00	42	17	4
	585.2852	-87.0	-148.7	16.5	C36 H41 O7	397.8	8.982	0.01	36	41	7
	585.1092	89.0	152.1	12.5	C24 H25 O17	394.3	5.448	0.43	24	25	17
	585.2911	-92.9	-158.7	7.5	C29 H45 O12	395.9	7.040	0.09	29	45	12
	585.1033	94.9	162.2	21.5	C31 H21 O12	397.2	8.300	0.02	31	21	12
	585.0998	98.4	168.1	-0.5	C13 H29 O25	394.7	5.882	0.28	13	29	25
	585.2970	-98.8	-168.8	-1.5	C22 H49 O17	421.4	32.534	0.00	22	49	17

Desktop 14:32

Spectrum - [170922_05]

File Edit Display Process Tools Window Help

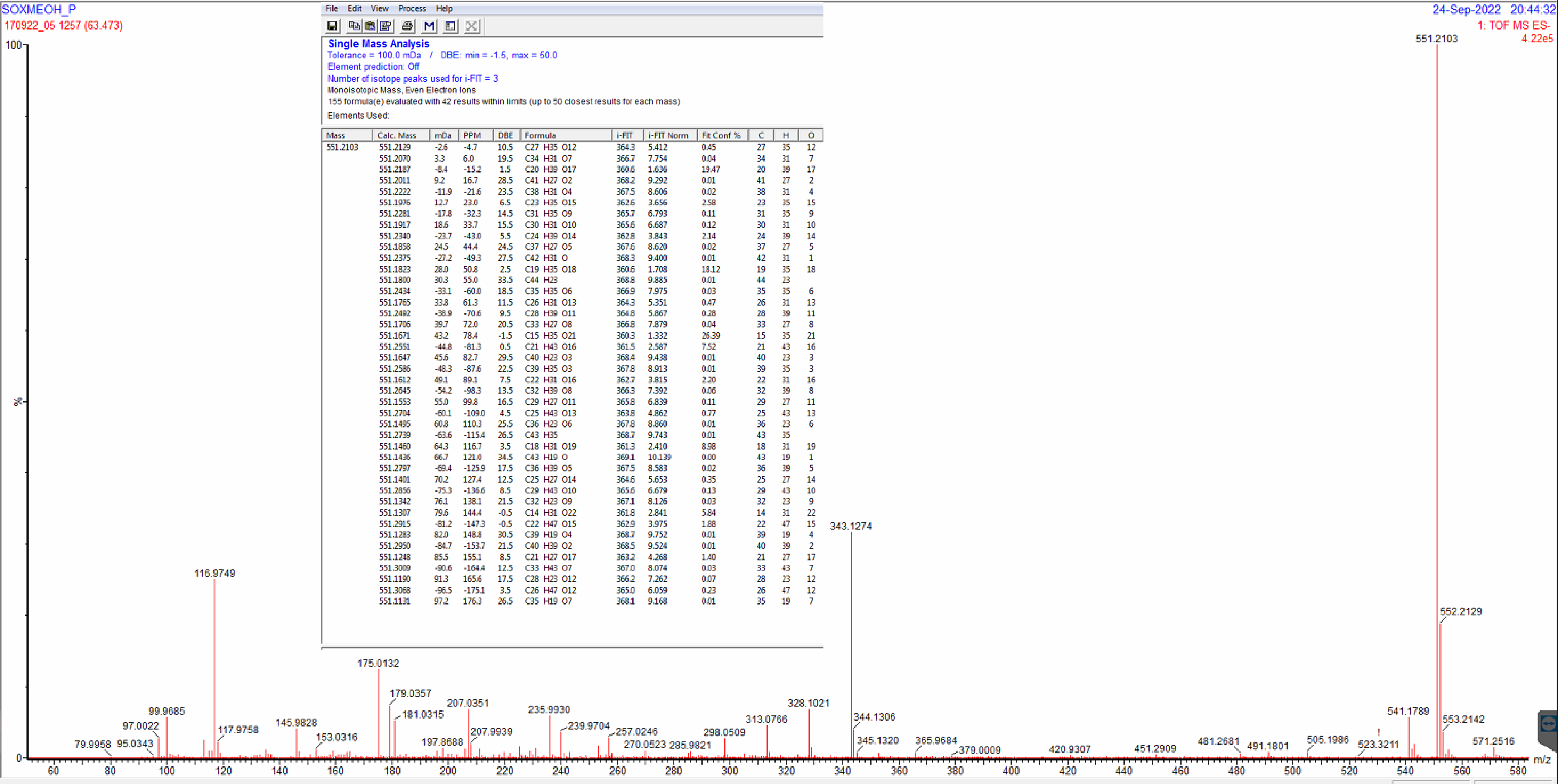

File Edit View Process Help

Single Mass Analysis

Tolerance = 100.0 mDa / DBE: min = -1.5, max = 50.0

Element prediction: Off

Number of isotope peaks used for i-FIT = 3

Monoisotopic Mass, Even Electron Ions

155 formula(e) evaluated with 42 results within limits (up to 50 closest results for each mass)

Elements Used:

Mass	Calc. Mass	mDa	PPM	DBE	Formula	i-FIT	i-FIT Norm	Fit Conf %	C	H	O
551.2103	551.2129	-2.6	-4.7	10.5	$C_{27} H_{35} O_{12}$	364.3	5.412	0.45	27	35	12
	551.2070	3.3	6.0	19.5	$C_{34} H_{31} O_{7}$	366.7	7.754	0.04	34	31	7
	551.2187	-8.4	-15.2	1.5	$C_{20} H_{39} O_{17}$	360.6	1.636	19.47	20	39	17
	551.2011	9.2	16.7	28.5	$C_{41} H_{27} O_{2}$	368.2	9.292	0.01	41	27	2
	551.2222	-11.9	-21.6	23.5	$C_{38} H_{31} O_{4}$	367.5	8.606	0.02	38	31	4
	551.1976	12.7	23.0	6.5	$C_{23} H_{35} O_{15}$	362.6	3.656	2.58	23	35	15
	551.2281	-17.8	-32.3	14.5	$C_{31} H_{35} O_{9}$	365.7	6.793	0.11	31	35	9
	551.1917	18.6	33.7	15.5	$C_{30} H_{31} O_{10}$	365.6	6.687	0.12	30	31	10
	551.2340	-23.7	-43.0	5.5	$C_{24} H_{39} O_{14}$	362.8	3.843	2.14	24	39	14
	551.1858	24.5	44.4	24.5	$C_{37} H_{27} O_{5}$	367.6	8.620	0.02	37	27	5
	551.2375	-27.2	-49.3	27.5	$C_{42} H_{31} O$	368.3	9.400	0.01	42	31	1
	551.1823	28.0	50.8	2.5	$C_{19} H_{35} O_{18}$	360.6	1.708	18.12	19	35	18
	551.1800	30.3	55.0	33.5	$C_{44} H_{23}$	368.8	9.885	0.01	44	23	
	551.2434	-33.1	-60.0	18.5	$C_{35} H_{35} O_{6}$	366.9	7.975	0.03	35	35	6
	551.1765	33.8	61.3	11.5	$C_{26} H_{31} O_{13}$	364.3	5.351	0.47	26	31	13
	551.2492	-38.9	-70.6	9.5	$C_{28} H_{39} O_{11}$	364.8	5.867	0.28	28	39	11
	551.1706	39.7	72.0	20.5	$C_{33} H_{27} O_{8}$	366.8	7.879	0.04	33	27	8
	551.1671	43.2	78.4	-1.5	$C_{15} H_{35} O_{21}$	360.3	1.332	26.39	15	35	21
	551.2551	-44.8	-81.3	0.5	$C_{21} H_{43} O_{16}$	361.5	2.587	7.52	21	43	16
	551.1647	45.6	82.7	29.5	$C_{40} H_{23} O_{3}$	368.4	9.438	0.01	40	23	3
	551.2586	-48.3	-87.6	22.5	$C_{39} H_{35} O_{3}$	367.8	8.913	0.01	39	35	3
	551.1612	49.1	89.1	7.5	$C_{22} H_{31} O_{16}$	362.7	3.815	2.20	22	31	16
	551.2645	-54.2	-98.3	13.5	$C_{32} H_{39} O_{8}$	366.3	7.392	0.06	32	39	8
	551.1553	55.0	99.8	16.5	$C_{29} H_{27} O_{11}$	365.8	6.839	0.11	29	27	11
	551.2704	-60.1	-109.0	4.5	$C_{25} H_{43} O_{13}$	363.8	4.862	0.77	25	43	13
	551.1495	60.8	110.3	25.5	$C_{36} H_{23} O_{6}$	367.8	8.860	0.01	36	23	6
	551.2739	-63.6	-115.4	26.5	$C_{43} H_{35}$	368.7	9.743	0.01	43	35	
	551.1460	64.3	116.7	3.5	$C_{18} H_{31} O_{19}$	361.3	2.410	8.98	18	31	19
	551.1436	66.7	121.0	34.5	$C_{43} H_{19} O$	369.1	10.139	0.00	43	19	1
	551.2797	-69.4	-125.9	17.5	$C_{36} H_{39} O_{5}$	367.5	8.583	0.02	36	39	5
	551.1401	70.2	127.4	12.5	$C_{25} H_{27} O_{14}$	364.6	5.653	0.35	25	27	14
	551.2856	-75.3	-136.6	8.5	$C_{29} H_{43} O_{10}$	365.6	6.679	0.13	29	43	10
	551.1342	76.1	138.1	21.5	$C_{32} H_{23} O_{9}$	367.1	8.126	0.03	32	23	9
	551.1307	79.6	144.4	-0.5	$C_{14} H_{31} O_{22}$	361.8	2.841	5.84	14	31	22
	551.2915	-81.2	-147.3	-0.5	$C_{22} H_{47} O_{15}$	362.9	3.975	1.88	22	47	15
	551.1283	82.0	148.8	30.5	$C_{39} H_{19} O_{4}$	368.7	9.752	0.01	39	19	4
	551.2950	-84.7	-153.7	21.5	$C_{40} H_{39} O_{2}$	368.5	9.524	0.01	40	39	2
	551.1248	85.5	155.1	8.5	$C_{21} H_{27} O_{17}$	363.2	4.268	1.40	21	27	17
	551.3009	-90.6	-164.4	12.5	$C_{33} H_{43} O_{7}$	367.0	8.074	0.03	33	43	7
	551.1190	91.3	165.6	17.5	$C_{28} H_{23} O_{12}$	366.2	7.262	0.07	28	23	12
	551.3068	-96.5	-175.1	3.5	$C_{26} H_{47} O_{12}$	365.0	6.059	0.23	26	47	12
	551.1131	97.2	176.3	26.5	$C_{35} H_{19} O_{7}$	368.1	9.168	0.01	35	19	7

Desktop 14:34

Spectrum - [170922_05]

File Edit Display Process Tools Window Help

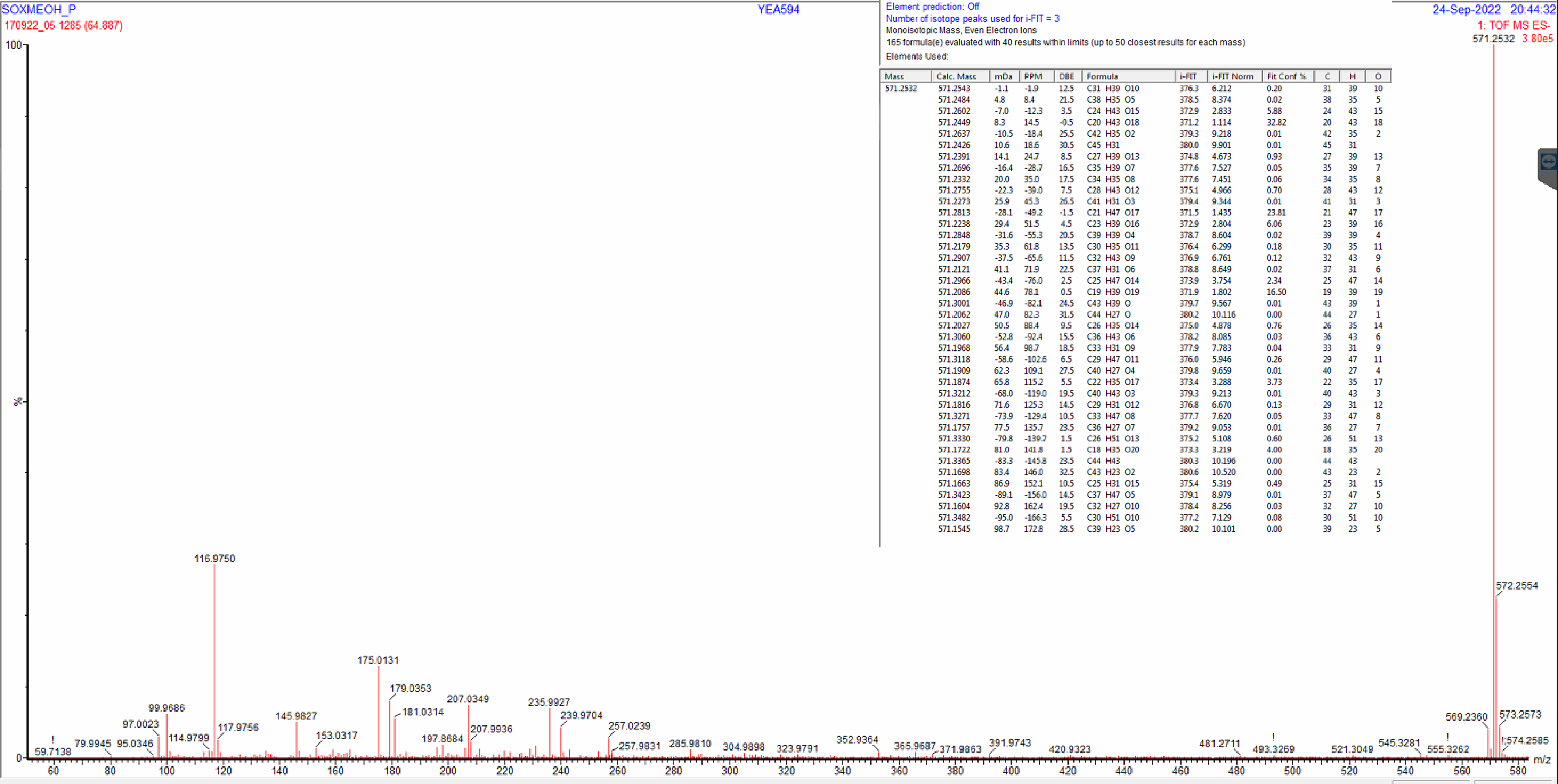

File Edit View Process Help

Single Mass Analysis

Tolerance = 100.0 mDa / DBE: min = -1.5, max = 50.0

Element prediction: Off

Number of isotope peaks used for i-FIT = 3

Monoisotopic Mass, Even Electron Ions

165 formula(e) evaluated with 40 results within limits (up to 50 closest results for each mass)

Elements Used:

Mass	Calc. Mass	mDa	PPM	DBE	Formula	i-FIT	i-FIT Norm	Fit Conf %	C	H	O
571.2532	571.2543	-1.1	-1.9	12.5	$C_{31}H_{39}O_{10}$	376.3	6.212	0.20	31	39	10
	571.2484	4.8	8.4	21.5	$C_{38}H_{35}O_{5}$	378.5	8.374	0.02	38	35	5
	571.2602	-7.0	-12.3	3.5	$C_{24}H_{43}O_{15}$	372.9	2.833	5.88	24	43	15
	571.2449	8.3	14.5	-0.5	$C_{20}H_{43}O_{18}$	371.2	1.114	32.82	20	43	18
	571.2637	-10.5	-18.4	25.5	$C_{42}H_{35}O_{2}$	379.3	9.218	0.01	42	35	2
	571.2426	10.6	18.6	30.5	$C_{45}H_{31}$	380.0	9.901	0.01	45	31	
	571.2391	14.1	24.7	8.5	$C_{27}H_{39}O_{13}$	374.8	4.673	0.93	27	39	13
	571.2696	-16.4	-28.7	16.5	$C_{35}H_{39}O_{7}$	377.6	7.527	0.05	35	39	7
	571.2332	20.0	35.0	17.5	$C_{34}H_{35}O_{8}$	377.6	7.451	0.06	34	35	8
	571.2755	-22.3	-39.0	7.5	$C_{28}H_{43}O_{12}$	375.1	4.966	0.70	28	43	12
	571.2273	25.9	45.3	26.5	$C_{41}H_{31}O_{3}$	379.4	9.344	0.01	41	31	3
	571.2813	-28.1	-49.2	-1.5	$C_{21}H_{47}O_{17}$	371.5	1.435	23.81	21	47	17
	571.2238	29.4	51.5	4.5	$C_{23}H_{39}O_{16}$	372.9	2.804	6.06	23	39	16
	571.2848	-31.6	-55.3	20.5	$C_{39}H_{39}O_{4}$	378.7	8.604	0.02	39	39	4
	571.2179	35.3	61.8	13.5	$C_{30}H_{35}O_{11}$	376.4	6.299	0.18	30	35	11
	571.2907	-37.5	-65.6	11.5	$C_{32}H_{43}O_{9}$	376.9	6.761	0.12	32	43	9
	571.2121	41.1	71.9	22.5	$C_{37}H_{31}O_{6}$	378.8	8.649	0.02	37	31	6
	571.2966	-43.4	-76.0	2.5	$C_{25}H_{47}O_{14}$	373.9	3.754	2.34	25	47	14
	571.2086	44.6	78.1	0.5	$C_{19}H_{39}O_{19}$	371.9	1.802	16.50	19	39	19
	571.3001	-46.9	-82.1	24.5	$C_{43}H_{39}O$	379.7	9.567	0.01	43	39	1
	571.2062	47.0	82.3	31.5	$C_{44}H_{27}O$	380.2	10.116	0.00	44	27	1
	571.2027	50.5	88.4	9.5	$C_{26}H_{35}O_{14}$	375.0	4.878	0.76	26	35	14
	571.3060	-52.8	-92.4	15.5	$C_{36}H_{43}O_{6}$	378.2	8.085	0.03	36	43	6
	571.1968	56.4	98.7	18.5	$C_{33}H_{31}O_{9}$	377.9	7.783	0.04	33	31	9
	571.3118	-58.6	-102.6	6.5	$C_{29}H_{47}O_{11}$	376.0	5.946	0.26	29	47	11
	571.1909	62.3	109.1	27.5	$C_{40}H_{27}O_{4}$	379.8	9.659	0.01	40	27	4
	571.1874	65.8	115.2	5.5	$C_{22}H_{35}O_{17}$	373.4	3.288	3.73	22	35	17
	571.3212	-68.0	-119.0	19.5	$C_{40}H_{43}O_{3}$	379.3	9.213	0.01	40	43	3
	571.1816	71.6	125.3	14.5	$C_{29}H_{31}O_{12}$	376.8	6.670	0.13	29	31	12
	571.3271	-73.9	-129.4	10.5	$C_{33}H_{47}O_{8}$	377.7	7.620	0.05	33	47	8
	571.1757	77.5	135.7	23.5	$C_{36}H_{27}O_{7}$	379.2	9.053	0.01	36	27	7
	571.3330	-79.8	-139.7	1.5	$C_{26}H_{51}O_{13}$	375.2	5.108	0.60	26	51	13
	571.1722	81.0	141.8	1.5	$C_{18}H_{35}O_{20}$	373.3	3.219	4.00	18	35	20
	571.3365	-83.3	-145.8	23.5	$C_{44}H_{43}$	380.3	10.196	0.00	44	43	
	571.1698	83.4	146.0	32.5	$C_{43}H_{23}O_{2}$	380.6	10.520	0.00	43	23	2
	571.1663	86.9	152.1	10.5	$C_{25}H_{31}O_{15}$	375.4	5.319	0.49	25	31	15
	571.3423	-89.1	-156.0	14.5	$C_{37}H_{47}O_{5}$	379.1	8.979	0.01	37	47	5
	571.1604	92.8	162.4	19.5	$C_{32}H_{27}O_{10}$	378.4	8.256	0.03	32	27	10
	571.3482	-95.0	-166.3	5.5	$C_{30}H_{51}O_{10}$	377.2	7.129	0.08	30	51	10
	571.1545	98.7	172.8	28.5	$C_{39}H_{23}O_{5}$	380.2	10.101	0.00	39	23	5

Desktop 14:39

Spectrum - [170922_05]

File Edit Display Process Tools Window Help

SOXMEOH_P YEA594 24-Sep-2022 20:44:32

170922_05 1549 (78.220) 1: TOF MS ES- 3.00e5

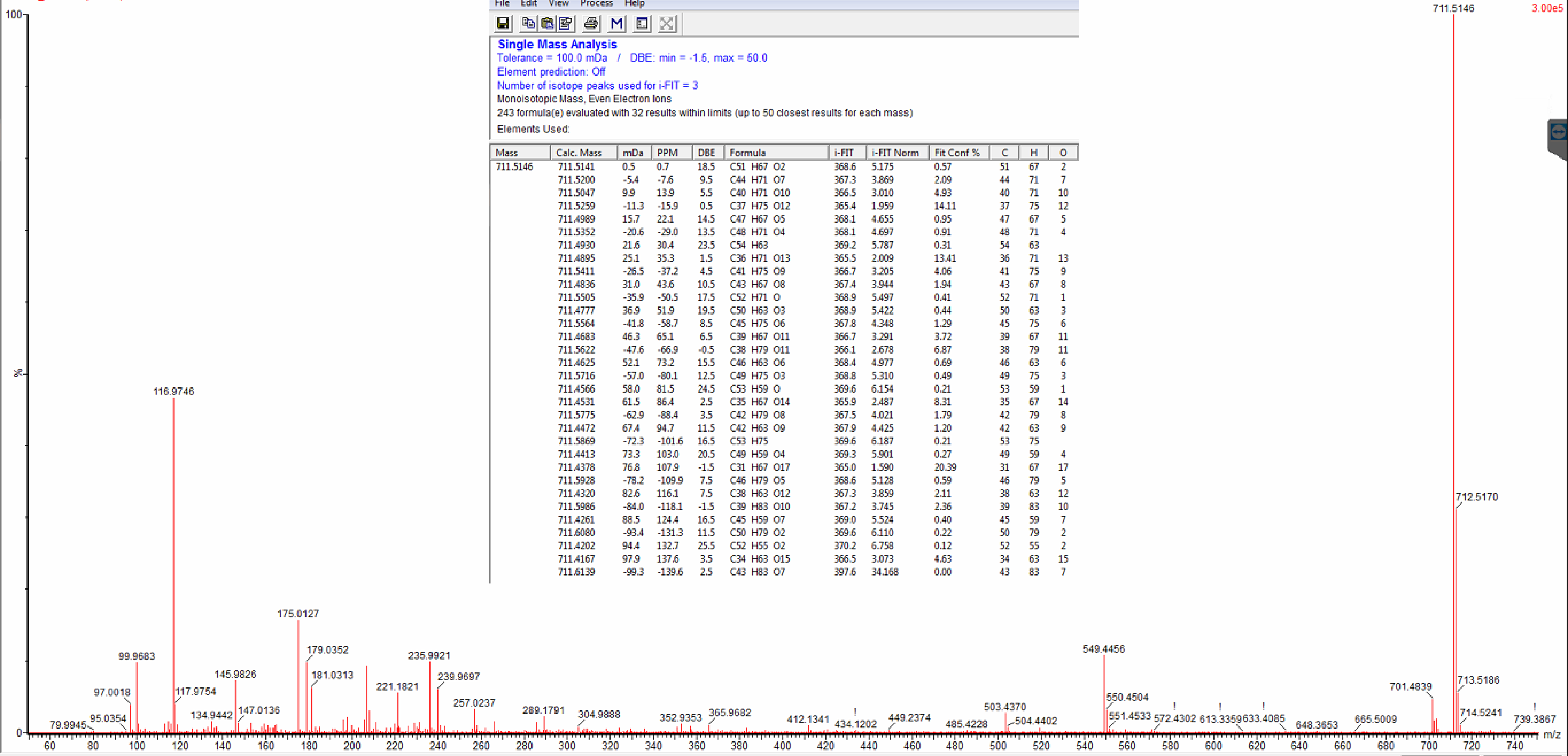

File Edit View Process Help

Single Mass Analysis

Tolerance = 100.0 mDa / DBE: min = -1.5, max = 50.0

Element prediction: Off

Number of isotope peaks used for i-FIT = 3

Monoisotopic Mass, Even Electron Ions

243 formula(e) evaluated with 32 results within limits (up to 50 closest results for each mass)

Elements Used:

Mass	Calc. Mass	mDa	PPM	DBE	Formula	i-FIT	i-FIT Norm	Fit Conf %	C	H	O
711.5146	711.5141	0.5	0.7	18.5	C51 H67 O2	368.6	5.175	0.57	51	67	2
	711.5200	-5.4	-7.6	9.5	C44 H71 O7	367.3	3.869	2.09	44	71	7
	711.5047	9.9	13.9	5.5	C40 H71 O10	366.5	3.010	4.93	40	71	10
	711.5259	-11.3	-15.9	0.5	C37 H75 O12	365.4	1.959	14.11	37	75	12
	711.4989	15.7	22.1	14.5	C47 H67 O5	368.1	4.655	0.95	47	67	5
	711.5352	-20.6	-29.0	13.5	C48 H71 O4	368.1	4.697	0.91	48	71	4
	711.4930	21.6	30.4	23.5	C54 H63	369.2	5.787	0.31	54	63	
	711.4895	25.1	35.3	1.5	C36 H71 O13	365.5	2.009	13.41	36	71	13
	711.5411	-26.5	-37.2	4.5	C41 H75 O9	366.7	3.205	4.06	41	75	9
	711.4836	31.0	43.6	10.5	C43 H67 O8	367.4	3.944	1.94	43	67	8
	711.5505	-35.9	-50.5	17.5	C52 H71 O	368.9	5.497	0.41	52	71	1
	711.4777	36.9	51.9	19.5	C50 H63 O3	368.9	5.422	0.44	50	63	3
	711.5564	-41.8	-58.7	8.5	C45 H75 O6	367.8	4.348	1.29	45	75	6
	711.4683	46.3	65.1	6.5	C39 H67 O11	366.7	3.291	3.72	39	67	11
	711.5622	-47.6	-66.9	-0.5	C38 H79 O11	366.1	2.678	6.87	38	79	11
	711.4625	52.1	73.2	15.5	C46 H63 O6	368.4	4.977	0.69	46	63	6
	711.5716	-57.0	-80.1	12.5	C49 H75 O3	368.8	5.310	0.49	49	75	3
	711.4566	58.0	81.5	24.5	C53 H59 O	369.6	6.154	0.21	53	59	1
	711.4531	61.5	86.4	2.5	C35 H67 O14	365.9	2.487	8.31	35	67	14
	711.5775	-62.9	-88.4	3.5	C42 H79 O8	367.5	4.021	1.79	42	79	8
	711.4472	67.4	94.7	11.5	C42 H63 O9	367.9	4.425	1.20	42	63	9
	711.5869	-72.3	-101.6	16.5	C53 H75	369.6	6.187	0.21	53	75	
	711.4413	73.3	103.0	20.5	C49 H59 O4	369.3	5.901	0.27	49	59	4
	711.4378	76.8	107.9	-1.5	C31 H67 O17	365.0	1.590	20.39	31	67	17
	711.5928	-78.2	-109.9	7.5	C46 H79 O5	368.6	5.128	0.59	46	79	5
	711.4320	82.6	116.1	7.5	C38 H63 O12	367.3	3.859	2.11	38	63	12
	711.5986	-84.0	-118.1	-1.5	C39 H83 O10	367.2	3.745	2.36	39	83	10
	711.4261	88.5	124.4	16.5	C45 H59 O7	369.0	5.524	0.40	45	59	7
	711.6080	-93.4	-131.3	11.5	C50 H79 O2	369.6	6.110	0.22	50	79	2
	711.4202	94.4	132.7	25.5	C52 H55 O2	370.2	6.758	0.12	52	55	2
	711.4167	97.9	137.6	3.5	C34 H63 O15	366.5	3.073	4.63	34	63	15
	711.6139	-99.3	-139.6	2.5	C43 H83 O7	397.6	34.168	0.00	43	83	7

Desktop 14:42

Spectrum - [170922_05]

File Edit Display Process Tools Window Help

SOXMEOH_P YEA594 24-Sep-2022 20:44:32

170922_05 1643 (82.967) 1: TOF MS ES- 4.32e5

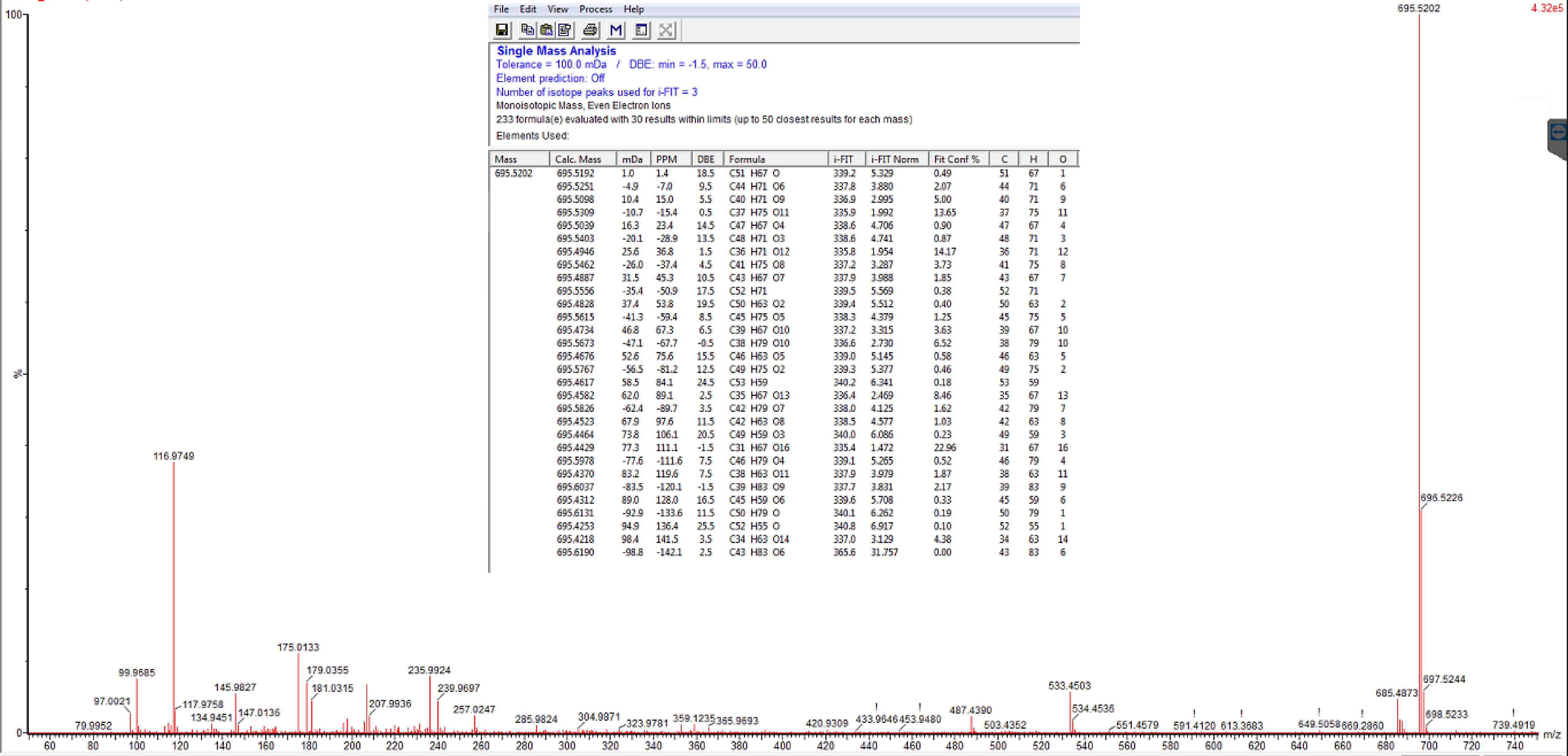

File Edit View Process Help

Single Mass Analysis

Tolerance = 100.0 mDa / DBE: min = -1.5, max = 50.0

Element prediction: Off

Number of isotope peaks used for i-FIT = 3

Monoisotopic Mass, Even Electron Ions

233 formula(e) evaluated with 30 results within limits (up to 50 closest results for each mass)

Elements Used:

Mass	Calc. Mass	mDa	PPM	DBE	Formula	i-FIT	i-FIT Norm	Fit Conf %	C	H	O
695.5202	695.5192	1.0	1.4	18.5	C51 H67 O	339.2	5.329	0.49	51	67	1
	695.5251	-4.9	-7.0	9.5	C44 H71 O6	337.8	3.880	2.07	44	71	6
	695.5098	10.4	15.0	5.5	C40 H71 O9	336.9	2.995	5.00	40	71	9
	695.5309	-10.7	-15.4	0.5	C37 H75 O11	335.9	1.992	13.65	37	75	11
	695.5039	16.3	23.4	14.5	C47 H67 O4	338.6	4.706	0.90	47	67	4
	695.5403	-20.1	-28.9	13.5	C48 H71 O3	338.6	4.741	0.87	48	71	3
	695.4946	25.6	36.8	1.5	C36 H71 O12	335.8	1.954	14.17	36	71	12
	695.5462	-26.0	-37.4	4.5	C41 H75 O8	337.2	3.287	3.73	41	75	8
	695.4887	31.5	45.3	10.5	C43 H67 O7	337.9	3.988	1.85	43	67	7
	695.5556	-35.4	-50.9	17.5	C52 H71	339.5	5.569	0.38	52	71	
	695.4828	37.4	53.8	19.5	C50 H63 O2	339.4	5.512	0.40	50	63	2
	695.5615	-41.3	-59.4	8.5	C45 H75 O5	338.3	4.379	1.25	45	75	5
	695.4734	46.8	67.3	6.5	C39 H67 O10	337.2	3.315	3.63	39	67	10
	695.5673	-47.1	-67.7	-0.5	C38 H79 O10	336.6	2.730	6.52	38	79	10
	695.4676	52.6	75.6	15.5	C46 H63 O5	339.0	5.145	0.58	46	63	5
	695.5767	-56.5	-81.2	12.5	C49 H75 O2	339.3	5.377	0.46	49	75	2
	695.4617	58.5	84.1	24.5	C53 H59	340.2	6.341	0.18	53	59	
	695.4582	62.0	89.1	2.5	C35 H67 O13	336.4	2.469	8.46	35	67	13
	695.5826	-62.4	-89.7	3.5	C42 H79 O7	338.0	4.125	1.62	42	79	7
	695.4523	67.9	97.6	11.5	C42 H63 O8	338.5	4.577	1.03	42	63	8
	695.4464	73.8	106.1	20.5	C49 H59 O3	340.0	6.086	0.23	49	59	3
	695.4429	77.3	111.1	-1.5	C31 H67 O16	335.4	1.472	22.96	31	67	16
	695.5978	-77.6	-111.6	7.5	C46 H79 O4	339.1	5.265	0.52	46	79	4
	695.4370	83.2	119.6	7.5	C38 H63 O11	337.9	3.979	1.87	38	63	11
	695.6037	-83.5	-120.1	-1.5	C39 H83 O9	337.7	3.831	2.17	39	83	9
	695.4312	89.0	128.0	16.5	C45 H59 O6	339.6	5.708	0.33	45	59	6
	695.6131	-92.9	-133.6	11.5	C50 H79 O	340.1	6.262	0.19	50	79	1
	695.4253	94.9	136.4	25.5	C52 H55 O	340.8	6.917	0.10	52	55	1
	695.4218	98.4	141.5	3.5	C34 H63 O14	337.0	3.129	4.38	34	63	14
	695.6190	-98.8	-142.1	2.5	C43 H83 O6	365.6	31.757	0.00	43	83	6

Desktop 14:45

Spectrum - [170922_05]

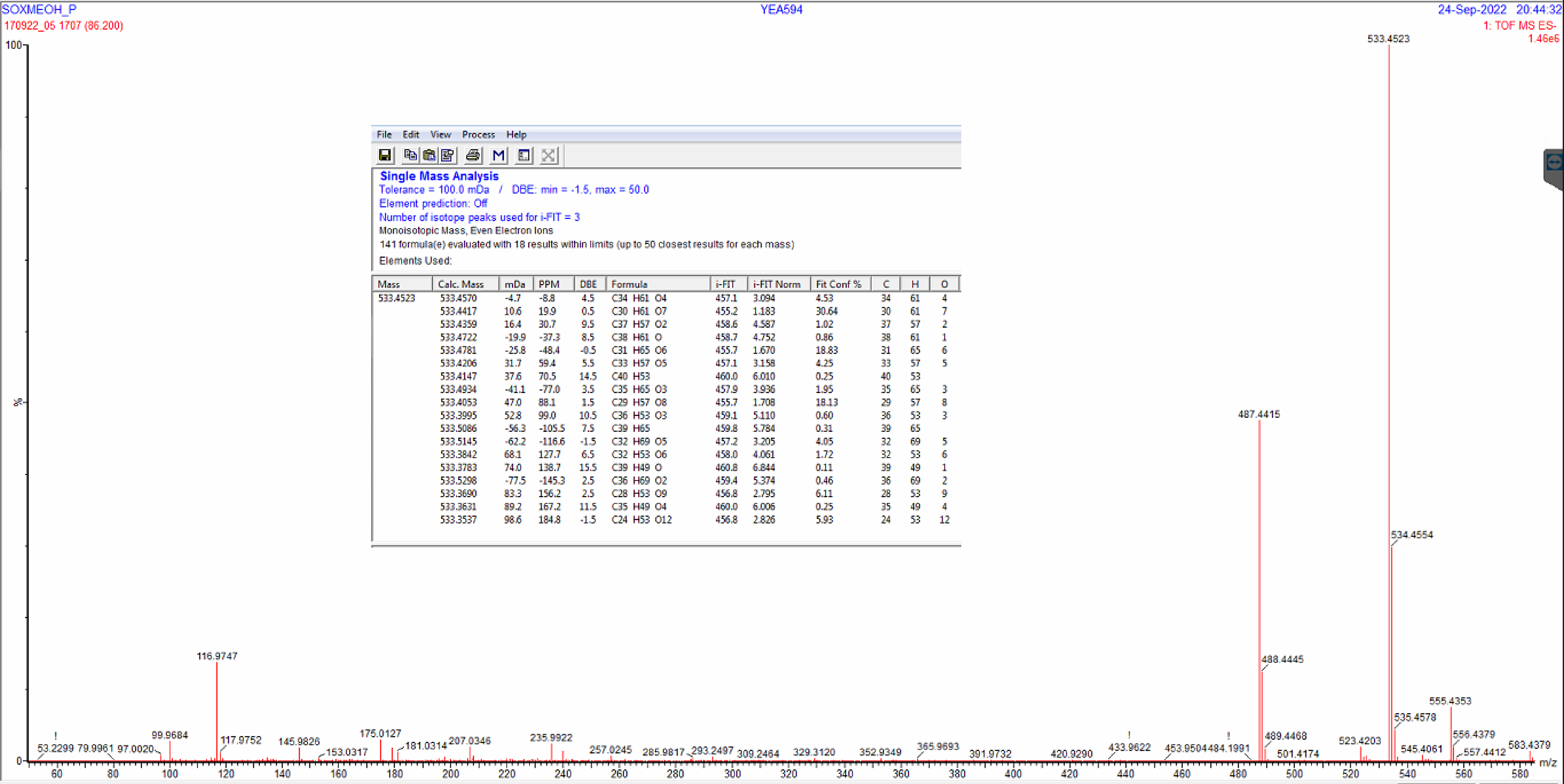

Single Mass Analysis

Tolerance = 100.0 mDa / DBE: min = -1.5, max = 50.0

Element prediction: Off

Number of isotope peaks used for i-FIT = 3

Monoisotopic Mass, Even Electron Ions

141 formula(e) evaluated with 18 results within limits (up to 50 closest results for each mass)

Elements Used:

Mass	Calc. Mass	mDa	PPM	DBE	Formula	i-FIT	i-FIT Norm	Fit Conf %	C	H	O
533.4523	533.4570	-4.7	-8.8	4.5	C34 H61 O4	457.1	3.094	4.53	34	61	4
	533.4417	10.6	19.9	0.5	C30 H61 O7	455.2	1.183	30.64	30	61	7
	533.4359	16.4	30.7	9.5	C37 H57 O2	458.6	4.587	1.02	37	57	2
	533.4722	-19.9	-37.3	8.5	C38 H61 O	458.7	4.752	0.86	38	61	1
	533.4781	-25.8	-48.4	-0.5	C31 H65 O6	455.7	1.670	18.83	31	65	6
	533.4206	31.7	59.4	5.5	C33 H57 O5	457.1	3.158	4.25	33	57	5
	533.4147	37.6	70.5	14.5	C40 H53	460.0	6.010	0.25	40	53	
	533.4934	-41.1	-77.0	3.5	C35 H65 O3	457.9	3.936	1.95	35	65	3
	533.4053	47.0	88.1	1.5	C29 H57 O8	455.7	1.708	18.13	29	57	8
	533.3995	52.8	99.0	10.5	C36 H53 O3	459.1	5.110	0.60	36	53	3
	533.5086	-56.3	-105.5	7.5	C39 H65	459.8	5.784	0.31	39	65	
	533.5145	-62.2	-116.6	-1.5	C32 H69 O5	457.2	3.205	4.05	32	69	5
	533.3842	68.1	127.7	6.5	C32 H53 O6	458.0	4.061	1.72	32	53	6
	533.3783	74.0	138.7	15.5	C39 H49 O	460.8	6.844	0.11	39	49	1
	533.5298	-77.5	-145.3	2.5	C36 H69 O2	459.4	5.374	0.46	36	69	2
	533.3690	83.3	156.2	2.5	C28 H53 O9	456.8	2.795	6.11	28	53	9
	533.3631	89.2	167.2	11.5	C35 H49 O4	460.0	6.006	0.25	35	49	4
	533.3537	98.6	184.8	-1.5	C24 H53 O12	456.8	2.826	5.93	24	53	12

Spectrum - [170922_05]

File Edit Display Process Tools Window Help

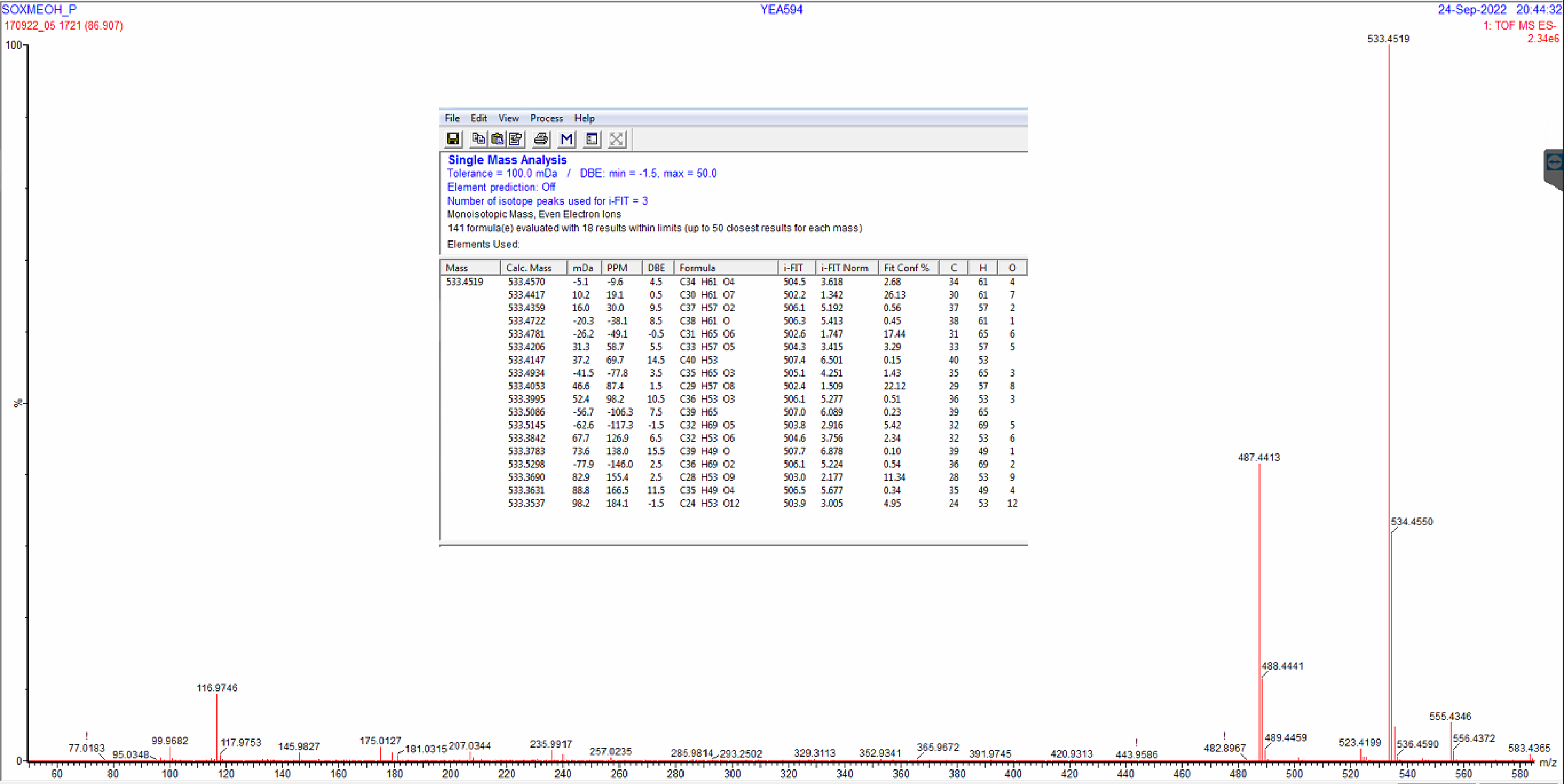

File Edit View Process Help

Single Mass Analysis

Tolerance = 100.0 mDa / DBE: min = -1.5, max = 50.0

Element prediction: Off

Number of isotope peaks used for i-FIT = 3

Monoisotopic Mass, Even Electron Ions

141 formula(e) evaluated with 18 results within limits (up to 50 closest results for each mass)

Elements Used:

Mass	Calc. Mass	mDa	PPM	DBE	Formula	i-FIT	i-FIT Norm	Fit Conf %	C	H	O
533.4519	533.4570	-5.1	-9.6	4.5	C34 H61 O4	504.5	3.618	2.68	34	61	4
	533.4417	10.2	19.1	0.5	C30 H61 O7	502.2	1.342	26.13	30	61	7
	533.4359	16.0	30.0	9.5	C37 H57 O2	506.1	5.192	0.56	37	57	2
	533.4722	-20.3	-38.1	8.5	C38 H61 O	506.3	5.413	0.45	38	61	1
	533.4781	-26.2	-49.1	-0.5	C31 H65 O6	502.6	1.747	17.44	31	65	6
	533.4206	31.3	58.7	5.5	C33 H57 O5	504.3	3.415	3.29	33	57	5
	533.4147	37.2	69.7	14.5	C40 H53	507.4	6.501	0.15	40	53	
	533.4934	-41.5	-77.8	3.5	C35 H65 O3	505.1	4.251	1.43	35	65	3
	533.4053	46.6	87.4	1.5	C29 H57 O8	502.4	1.509	22.12	29	57	8
	533.3995	52.4	98.2	10.5	C36 H53 O3	506.1	5.277	0.51	36	53	3
	533.5086	-56.7	-106.3	7.5	C39 H65	507.0	6.089	0.23	39	65	
	533.5145	-62.6	-117.3	-1.5	C32 H69 O5	503.8	2.916	5.42	32	69	5
	533.3842	67.7	126.9	6.5	C32 H53 O6	504.6	3.756	2.34	32	53	6
	533.3783	73.6	138.0	15.5	C39 H49 O	507.7	6.878	0.10	39	49	1
	533.5298	-77.9	-146.0	2.5	C36 H69 O2	506.1	5.224	0.54	36	69	2
	533.3690	82.9	155.4	2.5	C28 H53 O9	503.0	2.177	11.34	28	53	9
	533.3631	88.8	166.5	11.5	C35 H49 O4	506.5	5.677	0.34	35	49	4
	533.3537	98.2	184.1	-1.5	C24 H53 O12	503.9	3.005	4.95	24	53	12

Desktop 14:51

Spectrum - [170922_05]

File Edit Display Process Tools Window Help

File Edit View Process Help

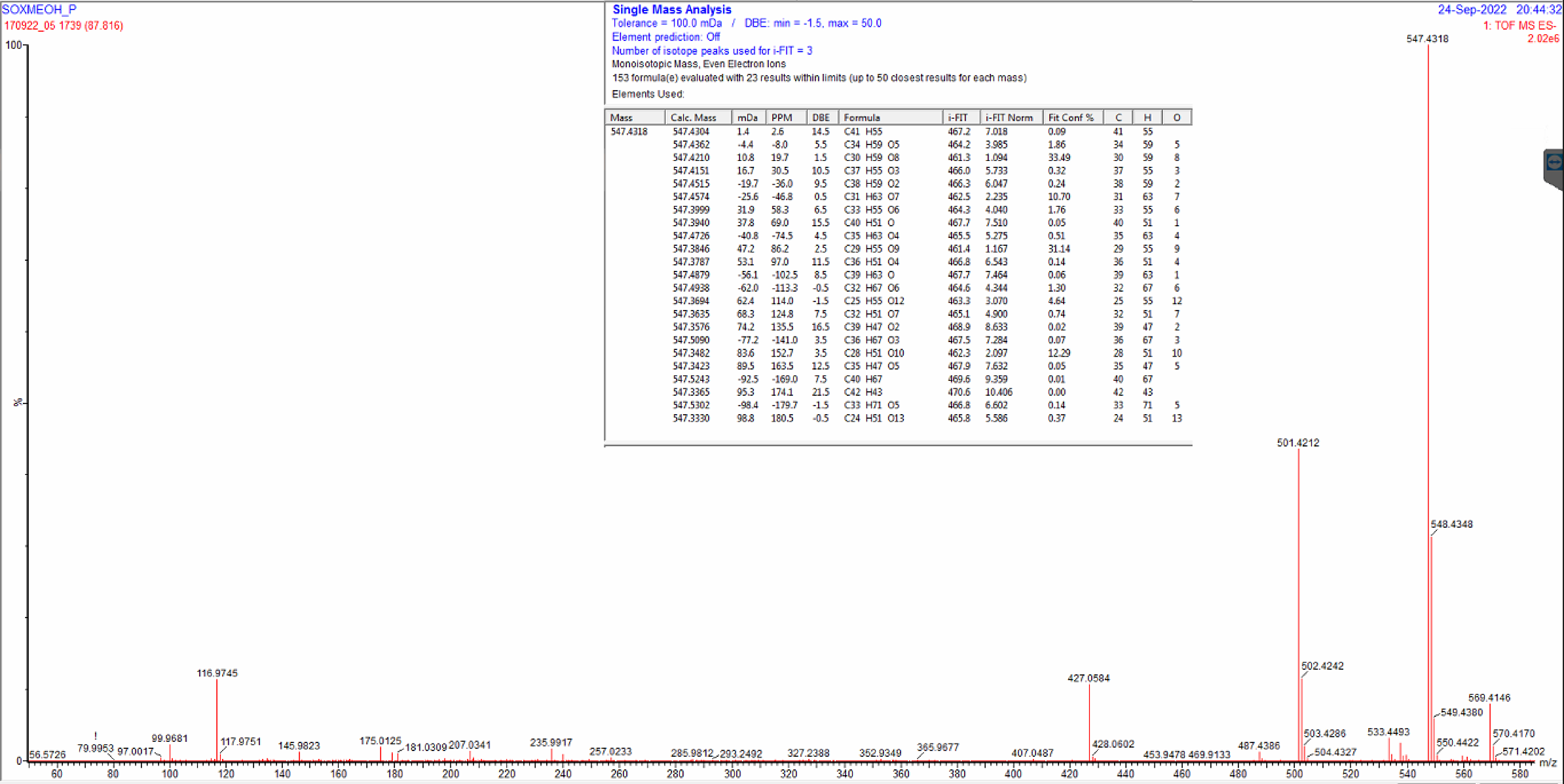

Single Mass Analysis

Tolerance = 100.0 mDa / DBE: min = -1.5, max = 50.0

Element prediction: Off

Number of isotope peaks used for i-FIT = 3

Monoisotopic Mass, Even Electron Ions

153 formula(e) evaluated with 23 results within limits (up to 50 closest results for each mass)

Elements Used:

Mass	Calc. Mass	mDa	PPM	DBE	Formula	i-FIT	i-FIT Norm	Fit Conf %	C	H	O
547.4318	547.4304	1.4	2.6	14.5	C41 H55	467.2	7.018	0.09	41	55	
	547.4362	-4.4	-8.0	5.5	C34 H59 O5	464.2	3.985	1.86	34	59	5
	547.4210	10.8	19.7	1.5	C30 H59 O8	461.3	1.094	33.49	30	59	8
	547.4151	16.7	30.5	10.5	C37 H55 O3	466.0	5.733	0.32	37	55	3
	547.4515	-19.7	-36.0	9.5	C38 H59 O2	466.3	6.047	0.24	38	59	2
	547.4574	-25.6	-46.8	0.5	C31 H63 O7	462.5	2.235	10.70	31	63	7
	547.3999	31.9	58.3	6.5	C33 H55 O6	464.3	4.040	1.76	33	55	6
	547.3940	37.8	69.0	15.5	C40 H51 O	467.7	7.510	0.05	40	51	1
	547.4726	-40.8	-74.5	4.5	C35 H63 O4	465.5	5.275	0.51	35	63	4
	547.3846	47.2	86.2	2.5	C29 H55 O9	461.4	1.167	31.14	29	55	9
	547.3787	53.1	97.0	11.5	C36 H51 O4	466.8	6.543	0.14	36	51	4
	547.4879	-56.1	-102.5	8.5	C39 H63 O	467.7	7.464	0.06	39	63	1
	547.4938	-62.0	-113.3	-0.5	C32 H67 O6	464.6	4.344	1.30	32	67	6
	547.3694	62.4	114.0	-1.5	C25 H55 O12	463.3	3.070	4.64	25	55	12
	547.3635	68.3	124.8	7.5	C32 H51 O7	465.1	4.900	0.74	32	51	7
	547.3576	74.2	135.5	16.5	C39 H47 O2	468.9	8.633	0.02	39	47	2
	547.5090	-77.2	-141.0	3.5	C36 H67 O3	467.5	7.284	0.07	36	67	3
	547.3482	83.6	152.7	3.5	C28 H51 O10	462.3	2.097	12.29	28	51	10
	547.3423	89.5	163.5	12.5	C35 H47 O5	467.9	7.632	0.05	35	47	5
	547.5243	-92.5	-169.0	7.5	C40 H67	469.6	9.359	0.01	40	67	
	547.3365	95.3	174.1	21.5	C42 H43	470.6	10.406	0.00	42	43	
	547.5302	-98.4	-179.7	-1.5	C33 H71 O5	466.8	6.602	0.14	33	71	5
	547.3330	98.8	180.5	-0.5	C24 H51 O13	465.8	5.586	0.37	24	51	13

Desktop 14:53
